# Genome‐wide association study in 21,271 individuals identifies 9 novel loci associated with circulating CD34^+^ hematopoietic stem and progenitor cell levels

**DOI:** 10.1002/hem3.70416

**Published:** 2026-07-01

**Authors:** Yuwen Xie, Antton Lamarca, Caterina Cafaro, Gudmar Thorleifsson, Ludvig Ekdahl, Daniela Torres Di Bello, Zain Ali, Yen La, Lilja Stefansdottir, Alex Antill, Maroulio Pertesi, Maria Laura Mahecha Escobar, Magnús K. Magnússon, Simon N. Stacey, Kari Stefansson, Ingileif Jonsdottir, Göran Karlsson, Thorunn Olafsdottir, Unnur Thorsteinsdottir, Aitzkoa Lopez de Lapuente Portilla, Björn Nilsson

**Affiliations:** ^1^ Lund Stem Cell Center Lund University Lund Sweden; ^2^ Department of Laboratory Medicine Lund University Lund Sweden; ^3^ Amgen deCODE Genetics Reykjavik Iceland; ^4^ Faculty of Medicine University of Iceland Reykjavik Iceland; ^5^ Division of Hematology Helsingborgs Lasarett Helsingborg Sweden; ^6^ Broad Institute Cambridge Massachusetts USA

Human hematopoiesis is sustained by hematopoietic stem and progenitor cells (HSPCs), which generate all mature blood cells. These cells reside primarily in the bone marrow, with a small fraction circulating in peripheral blood. Circulating HSPCs represent ~0.1% of white blood cells and are typically identified by expression of the surface marker CD34. The genetic mechanisms regulating circulating HSPC levels remain poorly understood. In a previous genome‐wide association study (GWAS) of 13,167 individuals, we identified 9 significant and 2 suggestive variants, explaining 4.6% of the variance.^1^ While these findings confirmed a genetic contribution, the broader architecture remains unclear. We therefore conducted an expanded GWAS and now identified 19 independent variants, 9 of which are novel.

We performed a meta‐analysis of 21,271 individuals of Swedish ancestry aged 18–71 years, including one dataset comprising 9627 new participants and one comprising 11,644 from our previous study^1^ (Supporting Information S3: Methods, Supporting Information S2: Table 1, and Supporting Information S1: Figure 1). Blood CD34^+^ cell levels were measured by flow cytometry, analyzing up to 1 million white blood cells per sample (median 562,688; Supporting Information S2: Tables 2 and 3) and defined as the fraction of CD34^+^ cells among CD45^+^ mononuclear cells (Supporting Information S1: Figure 2). To enable operator‐independent analysis of the large amounts of flow cytometry data, we developed bespoke software for automated pattern‐recognition gating (AliGater; Supporting Information S3: Methods). Participants were genotyped for 32 million single‐nucleotide polymorphisms (SNPs) and small insertions/deletions (INDELs) using microarrays and imputation. To account for multiple testing, we applied Bonferroni correction across five variant classes defined by genomic annotations (Supporting Information S2: Table 4). To identify independent variants, we used stepwise conditional analysis.

We identified 10 known and 9 novel associations (Figure 1A,B, Supporting Information S2: Table 5). Ten of the 11 associations from our previous study replicated at genome‐wide significance, with only the suggestive 5p15/*TERT* association not reaching this threshold (P = 5.36 × 10^−2^). We did not detect any heterogeneity in effect size estimates between the new and previously published sample sets (Supporting Information S2: Table 5). Lead variants explained 5.95% of phenotypic variance. Linkage disequilibrium score regression (LDSC) estimated the total SNP heritability at 27.8%. Partitioned heritability analysis using LDSC and g‐chromVAR revealed an enrichment in accessible chromatin of CD34^+^ cell types, indicating that the genetic regulation of blood CD34^+^ cell levels is partly mediated by cell‐autonomous effects in HSPCs, particularly of myeloid lineage (Figure 1C, Supporting Information S1: Figure 3).

**Figure 1:**
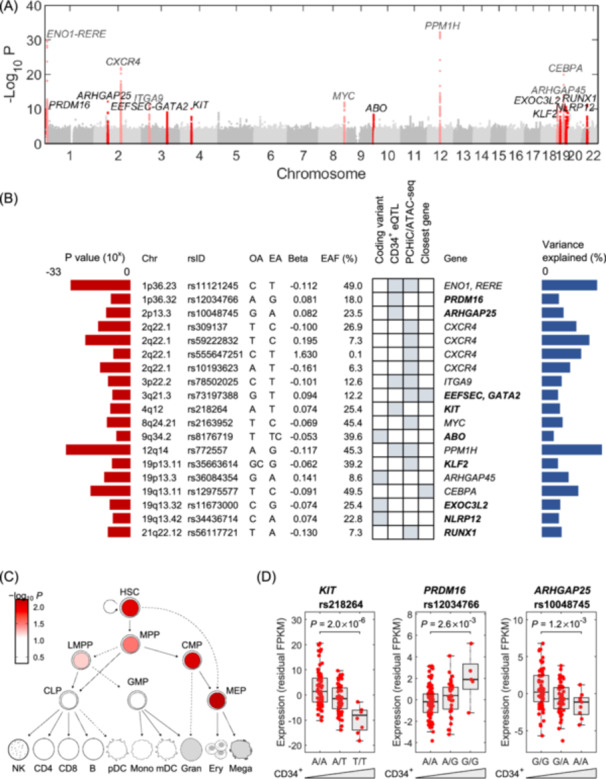
**Results of genome‐wide association study. (A)** Manhattan plot. The *x*‐axis indicates the genomic position of variants tested. The *y*‐axis indicates −log_10_ P‐value of association between variants and blood CD34^+^ cell levels. New loci indicated in black, previously reported loci in gray. **(B)** Lead variants of the 19 independent, genome‐significant association signals (Supporting Information S1: Figure 4 and Supporting Information S2: Table 5). Red bars indicate −log_10_ P‐value for association with blood CD34^+^ cell levels. Blue bars indicate the proportion of variance explained. Gene names indicate the candidate genes of each association (genes at novel loci in black; genes at previously reported loci in gray). **(C)** Linkage disequilibrium score regression showed enrichments of heritability in regions of accessible chromatin in HSPC subsets (red scale indicates −log_10_ P‐value). **(D)** *cis*‐eQTLs in sorted CD34^+^ cells from blood donors for the novel *KIT*, *PRDM16*, and *ARHGAP25* associations. Previously, we also identified *cis*‐eQTLs in CD34^+^ cells for the *PPM1H*, *ENO1‐RERE*, *ITGA9*, and three *CXCR4* associations. Data are residual FPKM values after correction for 10 expression principal components. Wedges indicate directions of effects on blood CD34^+^ cell levels for the same variant. CLP, common lymphoid progenitor; CMP, common myeloid progenitor; EA, effect allele; EAF, minor allele frequency; FPKM, fragments per kilobase of transcript per million mapped reads; GMP, granulocyte–monocyte progenitor; HSC, hematopoietic stem cell; LMPP, lympho‐myeloid primed progenitor; MEP, megakaryocyte–erythroid progenitors; MPP, multipotential progenitor; OA, other allele.

We defined a 99% credible set for each association (Supporting Information S2: Table 6) and prioritized candidate genes based on overlap with non‐synonymous coding variants; expression quantitative trait loci in CD34^+^ cells (Figure 1D)^1^; and gene promoter or regulatory elements interacting with the promoter, supported by ATAC‐seq and PCHi‐C data for CD34^+^ cells (Supporting Information S1: Figure 4). If no gene met these criteria, we assigned the closest relevant gene (*CEBPA* at 19q13.11; *EEFSEC* and *GATA2* at 3q21.3).

We nominated 18 candidate genes (Figure 1). These showed higher expression in HSPCs versus non‐HSPC cells, both in single‐cell mRNA‐seq data from 35,882 mononuclear cells (Supporting Information S1: Figure 5A; one‐sided Wilcoxon P = 1.2 × 10^−14^) and in bulk mRNA‐seq data from sorted blood cells (Supporting Information S1: Figure 5D; one‐sided Wilcoxon P = 1.5 × 10^−4^). To map expression within the CD34^+^ compartment, we analyzed single‐cell CITE‐seq data for 4,905 lineage‐negative bone marrow CD34^+^ cells, revealing preferential expression (median‐centered log_2_ ratio > 1) in specific CD34^+^ subpopulations for several candidate genes, including *KLF2*, *PRDM16*, and *NLRP12* in hematopoietic stem cells (HSCs); *GATA2* in megakaryocyte–erythroid progenitors (MEPs) and mast cell‐basophil progenitors (MBs); and *EXOC3L2* in MB, MEP, multipotential progenitors (MPP), and granulocyte–monocyte progenitors (GMP) (Supporting Information S1: Figures 5B,C, 6, and 7). We also found enrichment of hematopoietic phenotypes in knock‐out mice (12/18 genes = 67% vs. 27% in the genome; Fisher's exact test P = 8.2 × 10^−5^; Supporting Information S2: Table 7).

Investigating the contribution of the identified variants and genes to human diseases and traits, we observed a substantial overlap with mature blood cell traits in the GWAS catalog and FinnGen (Supporting Information S2: Tables 8 and 9). Consistent with this observation, and the enrichment of associated variants in open chromatin in myeloid HSPC populations, analysis of genetic correlations between blood CD34^+^ cell levels and mature blood cell traits revealed significant positive correlations with several myeloid traits and negative correlations with several lymphoid traits (Supporting Information S2: Table 10). Further supporting a link between CD34^+^ cell levels and myelopoiesis, seven candidate genes are implicated in Mendelian blood disorders (Supporting Information S2: Table 11), including familial acute myeloid leukemia (AML) (*CEBPA* and *GATA2*), MonoMAC syndrome (*GATA2*), and familial platelet disorder (*RUNX1*). Somatic lesions in *CEBPA, CXCR4*, *GATA2*, *KIT, MYC*, *PRDM16*, and *RUNX1* are linked to hematologic malignancies originating from HSPCs, including AML, myelodysplastic syndrome (MDS), and myeloproliferative diseases (MPNs). To assess this relationship systematically, we examined the overlap between candidate genes and somatic driver genes in 87 tumor types using the IntOGen and Mitelman databases, demonstrating a selective enrichment in AML (Fisher's exact test P = 3.5 × 10^−9^ across all candidate genes; P = 1.0 × 10^−5^ without *CEBPA* and *GATA2*, which were assigned based on proximity; Supporting Information S2: Table 12). Further supporting a role in malignant hematopoiesis, 10 candidate genes are essential in cell lines derived from HSPC‐related malignancies in DepMap (Supporting Information S2: Table 13). Collectively, these findings indicate a genetic overlap between blood CD34^+^ cell levels and myeloid hematologic malignancies, particularly AML.

Examining the biological functions of candidate genes, we noted that 16 of the 18 fall into three interconnected categories (Figure 2).

**Figure 2:**
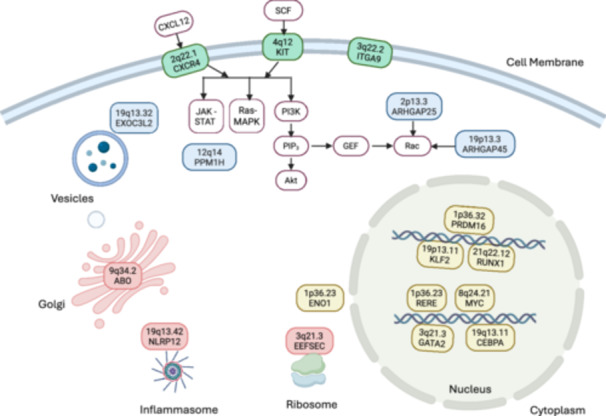
**Genes and pathways involved in the genetic regulation of blood CD34^+^ cell levels.** The boxes indicate candidate genes/proteins and the locus where they were identified. Box colors represent biological function: hematopoietic stem and progenitor cell (HSPC) surface receptors (green); intracellular signaling components (blue); hematopoietic transcription factors (yellow); and miscellaneous (pink). White boxes represent receptor ligands and signaling components with functional connections to candidate genes/proteins. This figure was created using BioRender. SCF, stem cell factor.

Six independent variants at three loci map to cell surface receptors directing HSPC trafficking_._ At 4q12, a novel association represented by rs218264 lies ~115 kb upstream of *KIT* (Proto‐oncogene c‐KIT). The variant lies in a region of accessible chromatin in HSPCs previously identified as a *KIT* enhancer in erythroid progenitors (Supporting Information S1: Figure 4).^2^ *KIT* encodes a receptor tyrosine kinase expressed in HSPCs (Supporting Information S1: Figure 5) that binds stem cell factor (SCF) and regulates several HSPC functions, including bone marrow retention.^3^ Elevated *KIT* expression drives HSPC differentiation with reduced blood CD34^+^ cell levels.^4^ Consistent with this model, rs218264‐T increases blood CD34^+^ cell levels while downregulating *KIT* in CD34^+^ cells (Figure 1D). Four previously identified variants at 2q22.1 (*CXCR4*) and one at 3p22.2 (*ITGA9*) also map to receptors with key roles in HSPC trafficking (Figure 1).^1^

Four variants map to genes involved in intracellular signaling, including downstream of CXCR4, KIT, and β1 integrins (one of which is ITGA9).

The novel 2p13.3 association represents a 5′‐UTR variant (rs10048745) at *ARHGAP25* (Rho‐Type GTPase‐Activating Protein 25). Rs10048745‐A confers higher blood CD34^+^ cell levels and downregulates *ARHGAP25* in CD34^+^ cells (Figure 1D). *ARHGAP25* is expressed in HSPCs (Supporting Information S1: Figure 5) and accelerates the GTP hydrolysis of Rac1 and Rac2 (Rac family small GTPase 1/2).^5^ Notably, conditional *Rac1* knockout reduces the ability of HSPCs to reconstitute hematopoiesis, and combined *Rac1/Rac2* knockout leads to massive HSPC mobilization.^6^ *Rac1* is activated by CXCL12, SCF, or integrin stimulation^7^ and *Arhgap25* ^−/−^ mice show increased HSPC marrow retention.^8^

At 19q13.32, rs11673000 (p.Arg98Pro) in *EXOC3L2* (Exocyst Complex Component 3 Like 2; Supporting Information S2: Table 6) is predicted to be deleterious (PolyPhen score 0; CADD score 24.6). *EXOC3L2* is expressed in HSPCs (Supporting Information S1: Figure 5). It encodes a homolog of EXOC3 in the exocyst complex (consisting of EXOC1 to 8) that anchors vesicles to the plasma membrane. Homozygous *EXOC3L2* mutations cause brain malformation renal syndrome (BMRS), which includes bone marrow failure.^9^ Hematological and endothelial knockout of *Exoc3l2* is embryonic lethal.^10^ The exocyst helps recycle surface receptors and interacts with small GTPases, notably Rac1 and RhoA, to couple vesicle trafficking to signaling.^11^ Induction of *EXOC3L2* in endothelial cells affects directional migration via EXOC4.^12^ Knockdown of *EXOC4* in hepatocarcinoma cells reduces CXCR4 surface presentation.^13^ These findings suggest that rs11673000 influences blood CD34^+^ cell levels by perturbing surface receptor trafficking and signaling, including through CXCR4.

Additionally, two known variants map to small GTPase modulators: *ARHGAP45* (rs36084354) catalyzes the GTP hydrolysis of Rac1 and RhoA; *PPM1H* (rs772557) encodes a serine phosphatase whose targets include Rab8a, implicated in CXCR4 trafficking.^14^, ^15^ These results implicate small‐GTPase signaling as a regulator of circulating HSPCs in humans.

Seven associations map to transcription factors with key roles in HSPC biology.

At 1p36.32, a novel association led by rs12034766 maps upstream of *PRDM16* (PR domain 16; Supporting Information S1: Figure 4). This gene contains histone lysine methyltransferase and zinc finger domains. It is expressed in HSPCs (Supporting Information S1: Figure 5) and regulates self‐renewal. Interestingly, gene fusions involving *PRDM16* occur in AML and MDS and result in its overexpression,^16^ and ectopic overexpression transforms murine progenitors into AML stem cells.^17^ Consistent with the oncogenic role of *PRDM16*, rs12034766‐G increases blood CD34^+^ cell levels while upregulating *PRDM16* (Figure 1D).

The 21q22.12 association maps to intron 1 of *RUNX1* (RUNX Family Transcription Factor 1), with the lead variant rs56117721 in accessible chromatin of HSPCs (Supporting Information S1: Figure 4). *RUNX1* encodes the α subunit of core binding factor, a master regulator of hematopoiesis. Germline *RUNX1* mutations cause familial platelet disorder with AML predisposition (Supporting Information S2: Table 11), while somatic lesions are recurrent in AML, MDS, and acute lymphoblastic leukemia. Presumably, rs56117721 affects blood cell CD34^+^ cell levels by altering *RUNX1* regulation.

At 3q21.3, rs73197388 tags a 177‐variant credible set at *EEFSEC* (Eukaryotic Elongation Factor, Selenocysteine‐tRNA Specific) near *GATA2* (GATA‐binding factor 2). *EEFSEC* is required for the synthesis of selenoproteins, which protect HSPCs from oxidative stress.^18^ *GATA2* encodes a transcription factor that regulates HSPC maintenance. Germline loss‐of‐function mutations in *GATA2* cause MonoMAC syndrome with bone marrow failure and AML predisposition (Supporting Information S2: Table 11).^19^ Somatic mutations are recurrent in AML. Several 3q21.3 variants are accessible in HSPCs with complex chromatin interactions (Supporting Information S1: Figure 4), suggesting they deregulate *EEFSEC*, *GATA2*, or both.

Additionally, rs35663614 maps to the 3′‐UTR of *KLF2* (Krüppel‐like factor 2), which has broad roles in hematopoiesis; and the known 19q13.11 (*CEPBA*), 1p36.23 (*ENO1‐RERE*), and 8q24.21 (*MYC*) associations map to hematopoietic transcription factors (*ENO1* encodes a glycolytic enzyme, but a secondary isoform interacts with MYC).^1^

The mechanisms regulating circulating HSPC levels in humans remain incompletely understood. We performed a GWAS of steady‐state blood CD34^+^ cell levels, enabled by high‐throughput flow cytometry, and identified 19 independent association signals, including 9 novel loci. A central finding is that the associated loci converge on three interconnected biological categories. The first consists of cell‐surface receptors directing stem cell trafficking and retention in the bone marrow (*KIT*, *CXCR4*, and *ITGA9*). The second comprises intracellular signaling pathways downstream of these receptors, particularly small GTPase modulators (*ARHGAP25*, *ARHGAP45*, *PPM1H*, and *EXOC3L2*). The third contains hematopoietic transcription factors that coordinate stem cell self‐renewal and lineage determination (*RUNX1*, *PRDM16*, and *GATA2*). Multiple independent loci map to these modules, revealing coordinated genetic regulation linking transcriptional programs, niche interactions, and intracellular signaling that govern HSPC migration and mobilization.

Sequence variants influencing blood CD34^+^ cell levels are enriched in open chromatin of HSPCs, particularly of myeloid lineage. Consistent with this, we identify positive genetic correlations and myeloid traits. Notably, several candidate genes (*RUNX1*, *GATA2*, and *CEBPA*) have been implicated in familial hematologic malignancies, particularly AML. Furthermore, we find a highly significant overlap between our set of candidate genes and genes that are recurrently somatically mutated in AML.

Our findings also highlight pathways that could be leveraged to improve stem cell mobilization. In stem cell transplantation, CD34^+^ cells are collected from donors via leukapheresis after mobilization with agents such as granulocyte colony‐stimulating factor (G‐CSF) and CXCR4 inhibitors. However, many donors remain poor mobilizers. Our previous discovery of *CXCR4* associations^1^ demonstrated that drug targets for stem cell mobilization can be identified through genetic variation, suggesting that other loci identified here may represent additional therapeutic opportunities. In particular, *ARHGAP25*, *ARHGAP45*, and *PPM1H* encode small GTPase modulators, and *EXOC3L2* links small GTPase signaling to receptor trafficking. Our results provide genetic support for the role of specific small‐GTPase modulators in regulating circulating CD34^+^ cell levels and motivate further studies to evaluate their potential as drug targets for stem cell mobilization.

In summary, we report a comprehensive analysis of the genetic architecture of blood CD34^+^ cell levels. Our results highlight variants and genes that regulate key aspects of HSPC biology, particularly stem cell trafficking, linking normal HSPC regulation with mechanisms underlying stem cell mobilization and malignant hematopoiesis.

## AUTHOR CONTRIBUTIONS

**Yuwen Xie**: Conceptualization; investigation; methodology; visualization; formal analysis; data curation; writing—original draft. **Antton Lamarca**: Investigation; methodology; software; data curation. **Caterina Cafaro**: Investigation. **Gudmar Thorleifsson**: Investigation; formal analysis; data curation; writing—review and editing. **Ludvig Ekdahl**: Investigation; formal analysis; methodology; software. **Daniela Torres Di Bello**: Methodology; investigation. **Zain Ali**: Investigation. **Yen La**: Investigation. **Lilja Stefansdottir**: Formal analysis; resources; investigation. **Alex Antill**: Resources; formal analysis. **Maroulio Pertesi**: Methodology; investigation. **Maria Laura Mahecha Escobar**: Investigation; methodology. **Magnús K. Magnússon**: Resources. **Simon N. Stacey**: Investigation; resources; writing—review and editing. **Kari Stefansson**: Resources. **Ingileif Jonsdottir**: Resources; writing—review and editing. **Göran Karlsson**: Resources. **Thorunn Olafsdottir**: Resources; writing—review and editing. **Unnur Thorsteinsdottir**: Funding acquisition; conceptualization; methodology; resources; writing—review and editing. **Aitzkoa Lopez de Lapuente Portilla**: Conceptualization; investigation; writing—original draft; writing—review and editing; methodology; validation; formal analysis; supervision; data curation. **Björn Nilsson**: Conceptualization; writing—original draft; investigation; visualization; writing—review and editing; software; supervision; formal analysis; project administration; funding acquisition.

## CONFLICT OF INTEREST STATEMENT

G.T., I.J., L.S., S.N.S., T.O., and U.T. are employed by Amgen deCODE Genetics. The remaining authors have no conflicts of interest.

## FUNDING

This work was supported by grants from the European Research Council (CoG‐770992 BloodVariome and EU‐MSCA‐COFUND 754299 CanFaster), the Knut and Alice Wallenberg Foundation (2014.0071 and 2017.0436), the Swedish Research Council (2017‐02023, 2018‐00424, and 2024‐02765), the Swedish Cancer Society (20.0694 and 23‐2851), the Swedish Children's Cancer Fund (PR2018‐0118 and PR2023‐0067), ARMEC Lindeberg's Foundation, Inga‐Britt and Arne Lundberg's Foundation (2017‐0055), and Region Skåne (ALF 2022‐0363).

## Supporting information

Supplementary Information.

Supplementary Information.

Supplementary Information.

## Data Availability

Summary statistics have been deposited in the GWAS Catalog under accession ID: GCST90828088 and will be made publicly available upon publication. The data that support the findings of this study are available from the corresponding author upon reasonable request.
