# Supplementary material for: Genome‐wide association study in 21,271 individuals identifies 9 novel loci associated with circulating CD34+ hematopoietic stem and progenitor cell levels
Source: Hemasphere. 2026 Jul 1;10(7):e70416. doi: 10.1002/hem3.70416 (PMC13321468; doi:10.1002/hem3.70416)
Supplement: Supplementary file 1 — Supplementary Information. [file HEM3-10-e70416-s002.pdf]

Supplementary Figure 1

Age distribution among the study participants.

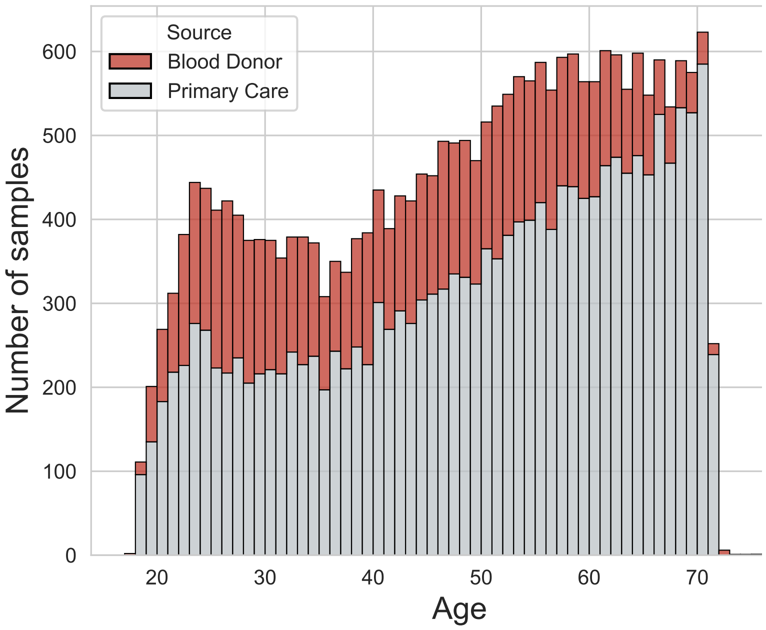

Supplemental Figure 2

Quantification of the $CD34^{+}$ cell frequency in blood. In each sample, we analyzed up to 1 million cells using flow cytometry. **(a)** First, we gated single cells based on forward scatter area and forward scatter height. **(b)** Second, we gated mononuclear cells based on side scatter area and forward scatter area. **(c)** Third, we gated $CD34^{+}$ cells (solid red) and $CD45^{+}$ cells (dashed red). $CD34^{+}$ cells form a cluster in the CD34-CD45 plot. We defined the blood $CD34^{+}$ cell level as the number of $CD34^{+}$ cells divided by the number of $CD45^{+}$ cells.

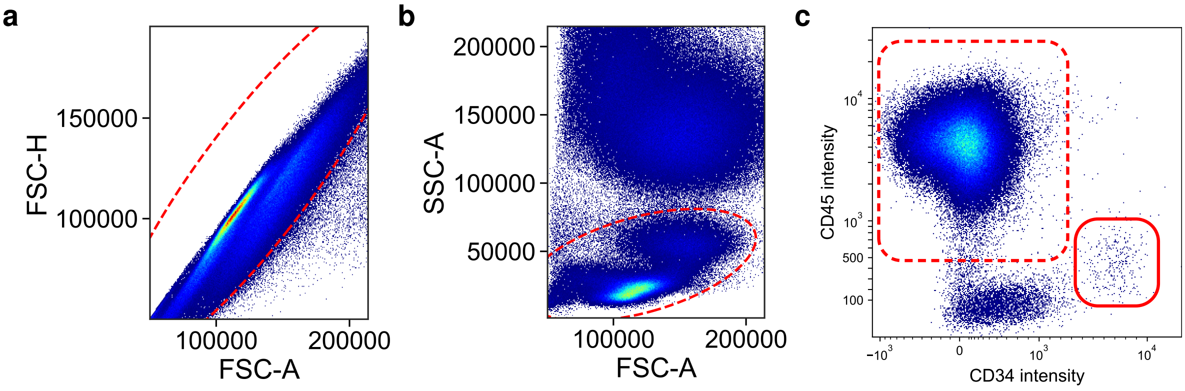

Supplementary Figure 3

Enrichments of heritability in regions of accessible chromatin in HSPC subsets as determined using g-chromVAR (red scale indicates -log10 P-value).

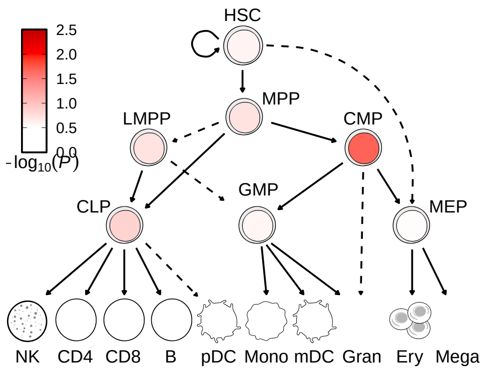

Supplementary Figure 4

Locus plots for the 19 significant associations with blood CD34$^+$ cell levels. Each dot represents a DNA sequence variant in a credible set at that locus. The x-axis indicates genomic position. The y-axis in the upper panels indicates statistical significance of the association with blood CD34$^+$ cell levels. The middle panel shows chromatin looping interactions, as determined using promoter capture Hi-C (PCHi-C) in isolated blood CD34$^+$ cells. Loops involving restriction fragments containing credible set variants are highlighted in red. The lower panels show chromatin accessibility in sorted CD34$^+$ blood cell types, with the y axis indicating ATAC-sequencing signal intensity. Abbreviations: hematopoietic stem cell (HSC), multi-potent progenitor (MPP), lympho-myeloid-primed progenitor (LMPP), common lymphoid progenitor (CLP), common myeloid progenitor (CMP), granulocyte-monocyte progenitor (GMP), and megakaryocyte-erythroid progenitor (MEP).

1p36.23 *ENO1*

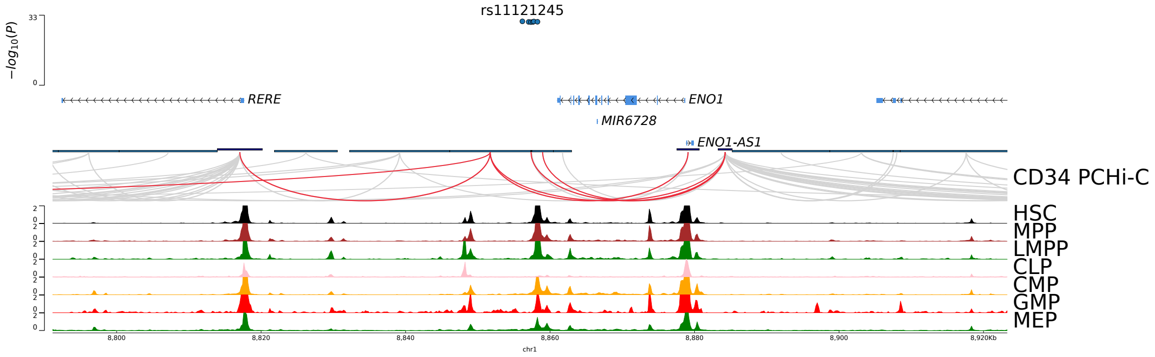

1p36.32 - *PRDM16*

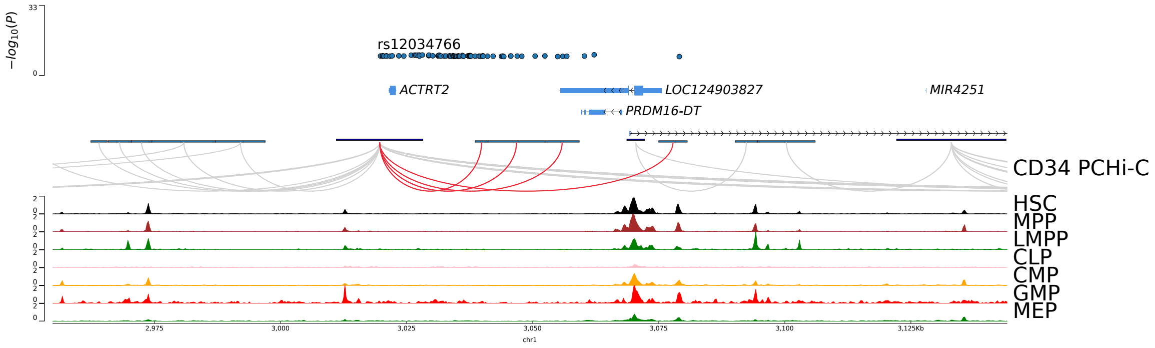

2p13.3 - *ARHGAP25*

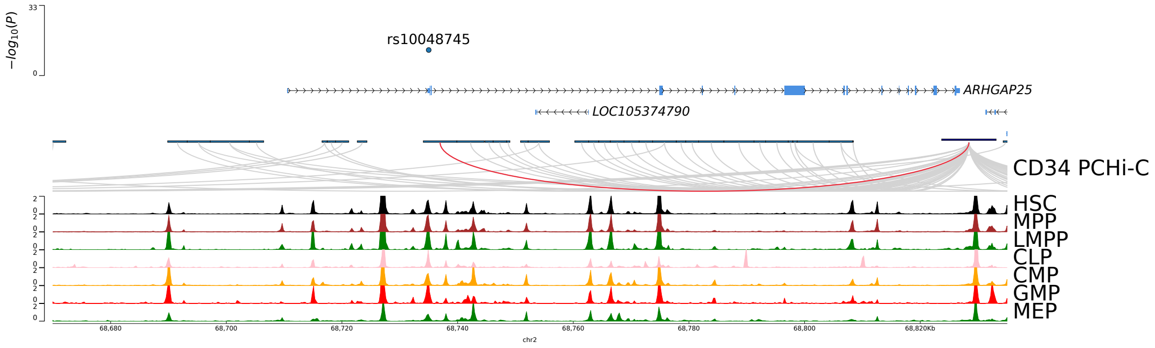

2q22.1 - *CXCR4*

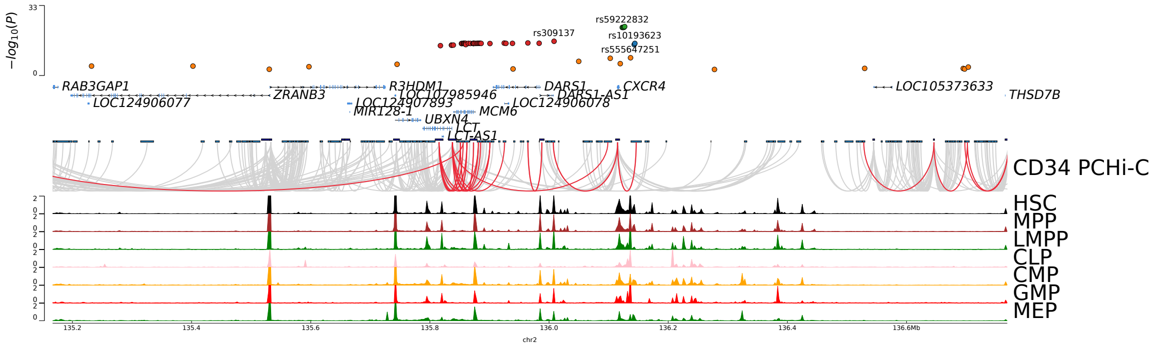

3p22.2 - *ITGA9*

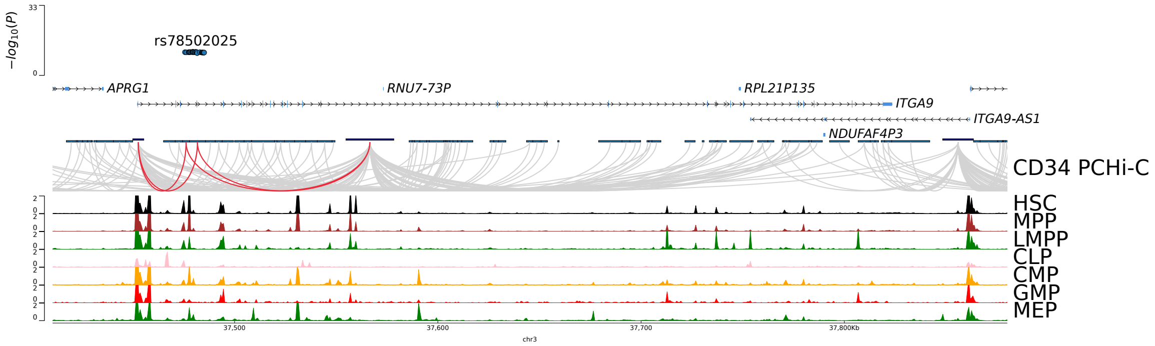

3q21.3 - *EEFSEC*, *GATA2*

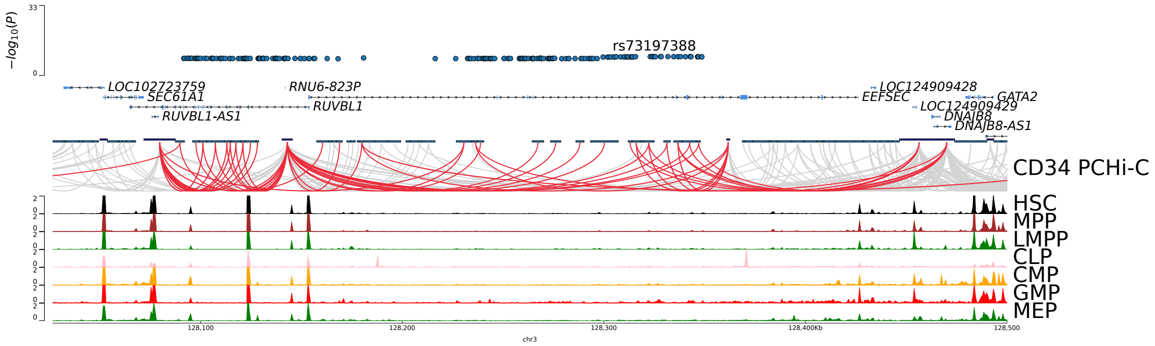

4q12 - *KIT*

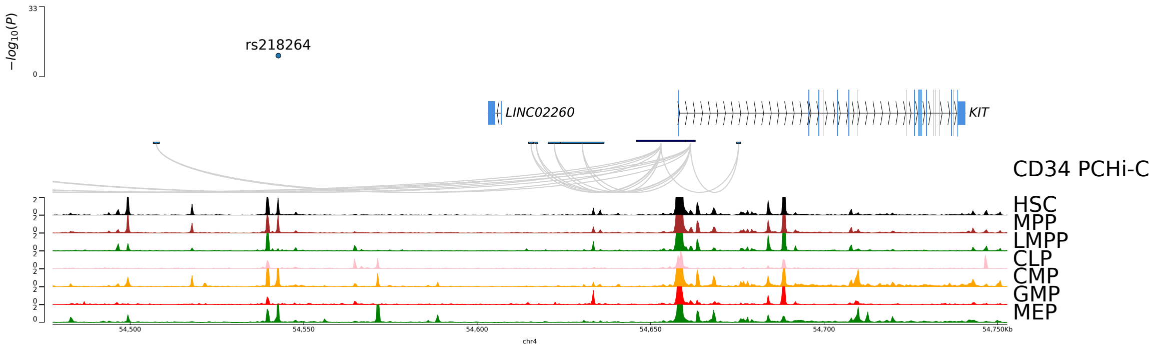

8q24.21 - *MYC*

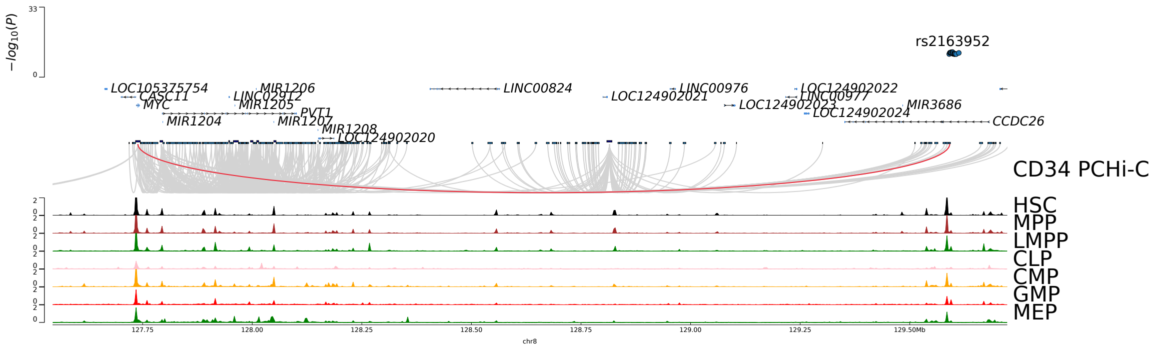

9q34.2 - *ABO*

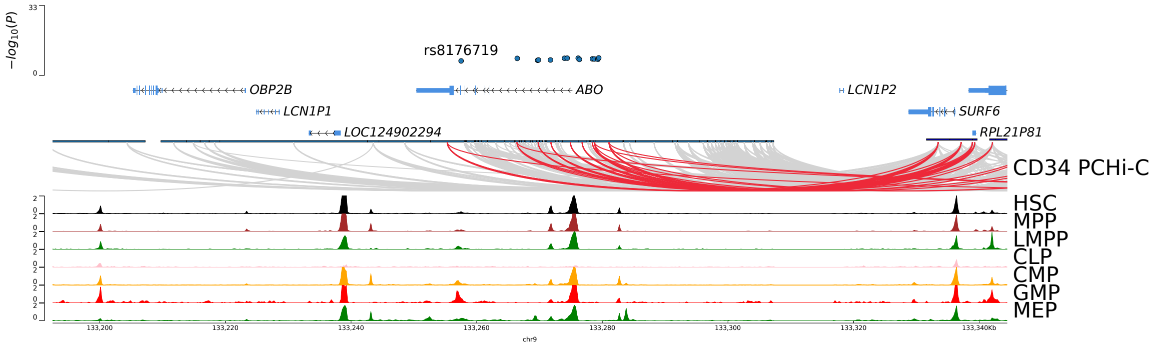

12q14 - *PPM1H*

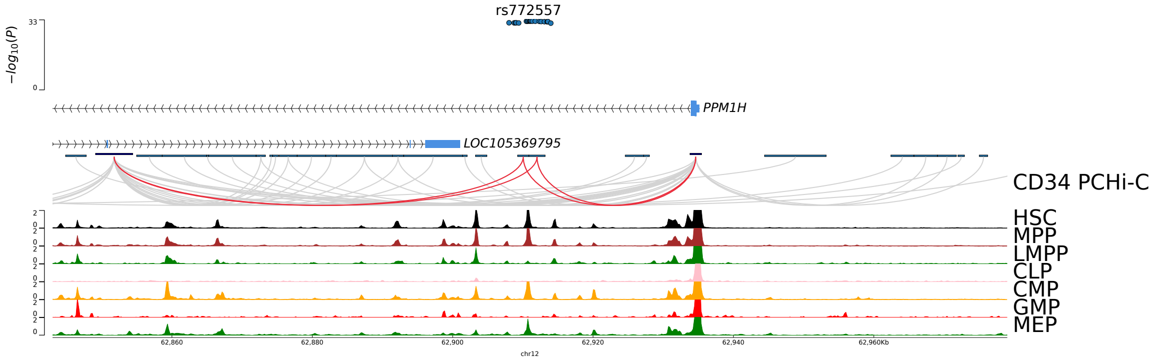

19p13.11 - *KLF2*

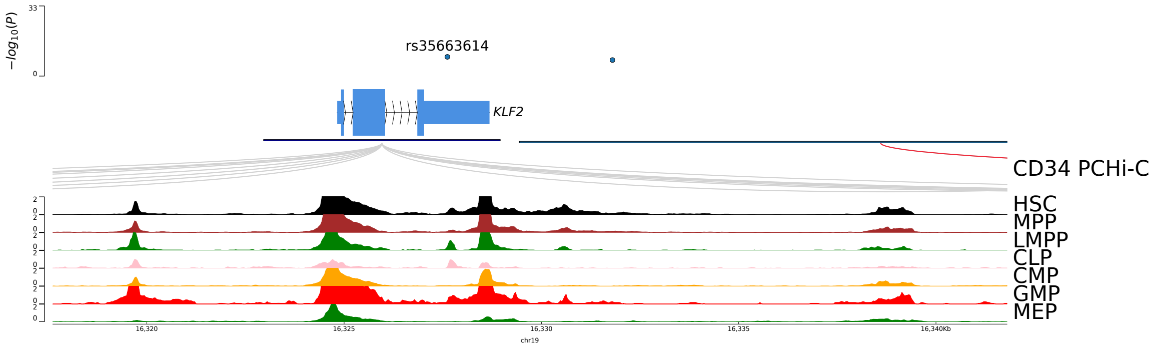

19p13.3 - *ARHGAP45*

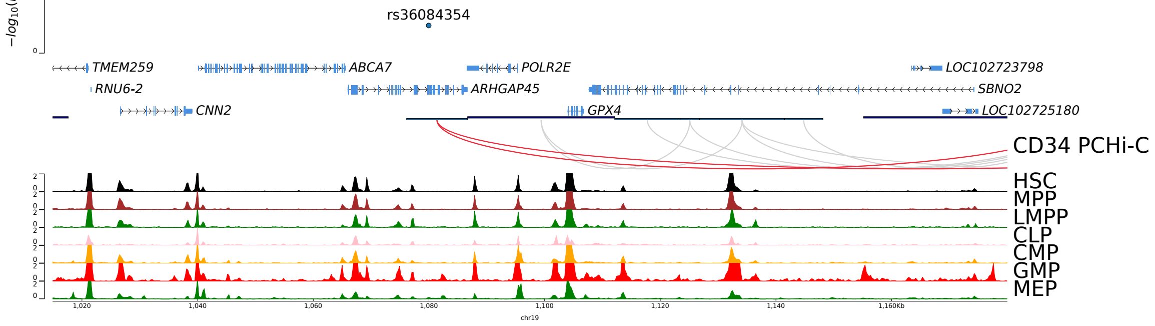

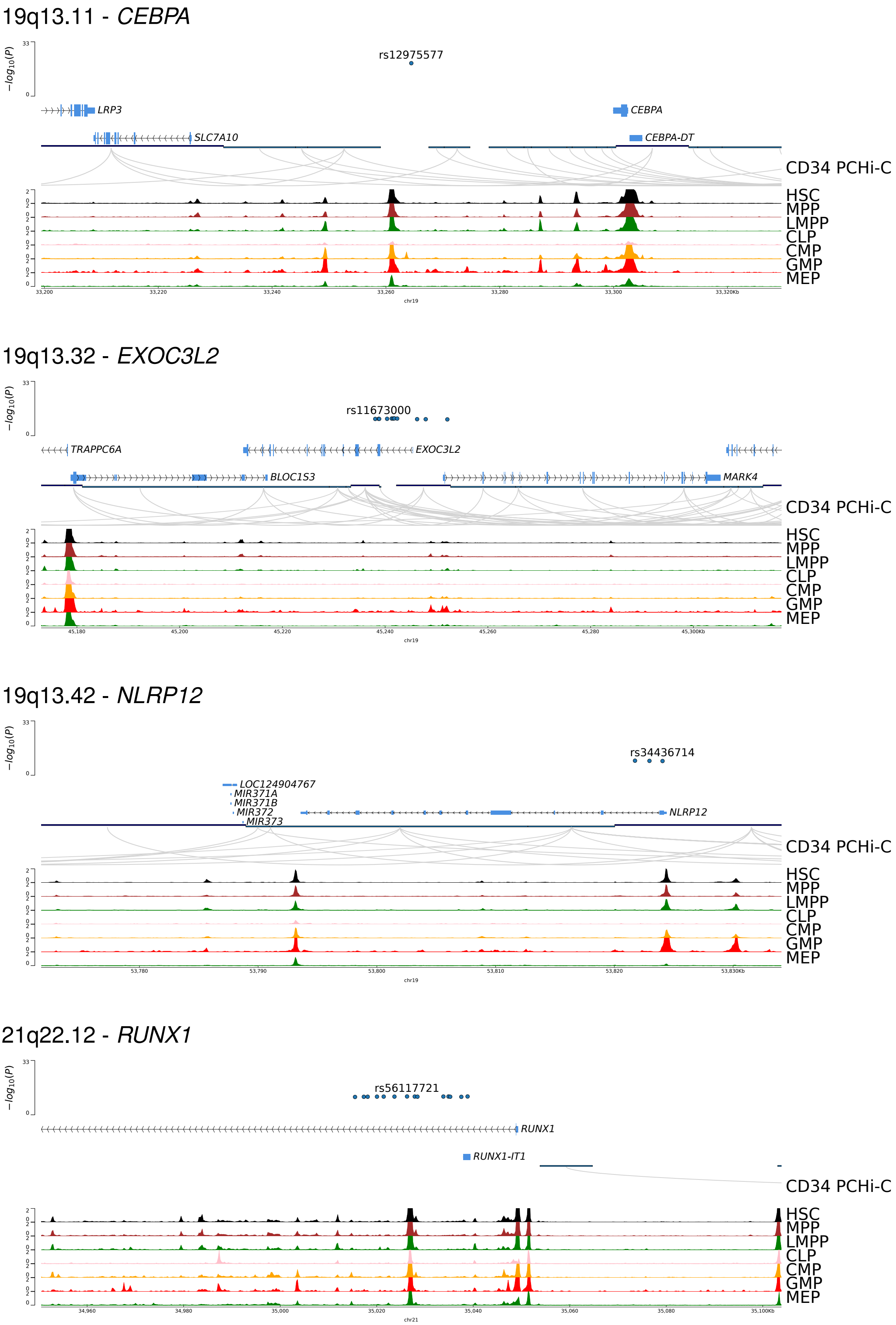
19q13.11 - CEBPA
rs12975577
LRP3
SLC7A10
CEBPA
CEBPA-DT
CD34 PCHi-C
HSC
MPP
LMPP
CLP
CMP
GMP
MEP
chr19
19q13.32 - EXOC3L2
rs11673000
TRAPPC6A
EXOC3L2
BLOC1S3
MARK4
CD34 PCHi-C
HSC
MPP
LMPP
CLP
CMP
GMP
MEP
chr19
19q13.42 - NLRP12
rs34436714
LOC124904767
MIR371A
MIR371B
MIR372
MIR373
NLRP12
CD34 PCHi-C
HSC
MPP
LMPP
CLP
CMP
GMP
MEP
chr19
21q22.12 - RUNX1
rs56117721
RUNX1
RUNX1-IT1
CD34 PCHi-C
HSC
MPP
LMPP
CLP
CMP
GMP
MEP
chr21

Supplementary Figure 5

Candidate gene expression across hematopoiesis. **(a)** scRNA-seq data from 35,582 blood and bone marrow mononuclear cells (Granja *et al.*, PMID 31792411). **(b,c)** Expression of candidate genes across different cell clusters in scCITE-seq data for 4,905 lineage-negative CD34$^+$ cells from adult bone marrow. In **(b)**, the 4,905 cells have been clustered by RNA-seq pattern. In **(c)**, the cells were instead clustered by gating using the sequence counts for the tags derived from antibodies to the CD38, CD45RA, CD90, CD123, and CD10 cell surface markers, as indicated at the upper edge of the heatmap. **(d)** Bulk mRNA-seq data of sorted blood cell types (Ulirsch *et al.*, PMID 30858613). Data are $\log_2$-transformed, median-centered expression values. Abbreviations: Hematopoietic stem cells (HSC), multi-potent progenitors (MPP), common myeloid progenitors (CMP), granulocyte-monocyte progenitors (GMP), common lymphoid progenitors (CLP), lymphoid-primed multipotent progenitors (LMPP), erythroid progenitors (ERY), megakaryocyte-erythrocyte progenitors (MEP), mast cell/basophil progenitors, (MB), dendritic cells (DC), plasma cells (PC), CD4+ T-cells (CD4), CD8+ T-cells (CD8), B-cells (B), pre B-cells (PreB), lymphoid progenitors (Ly), natural killer cells (NK), basophil (Baso), neutrophil (Neut), monocyte (Mono), cycling cells (Cyc).

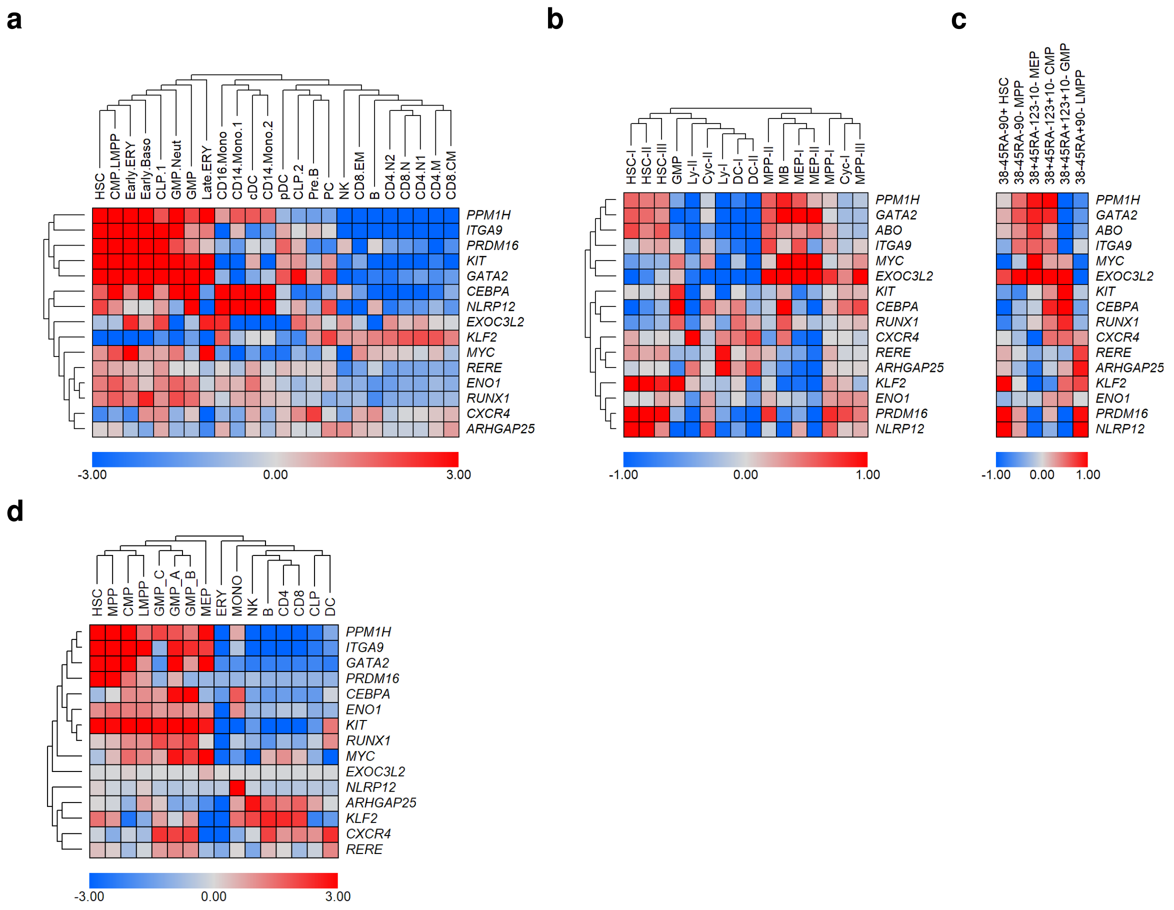

Supplementary Figure 6

Single-cell mRNA-seq data for 35,582 mononuclear cells from blood and bone marrow. Data from Granja *et al.* (Nature Biotechnology, 2019 Dec;37(12):1458-1465). Dimension-reduction using uniform manifold approximation and projection. The x- and y-axes indicate the projection of the expression pattern of each cell along the first and second UMAP components, shown per individual cell (left) or cell cluster (right). *ARHGAP45*, *CEBPA*, and *KIT* were not represented in this data set.

ARHGAP25

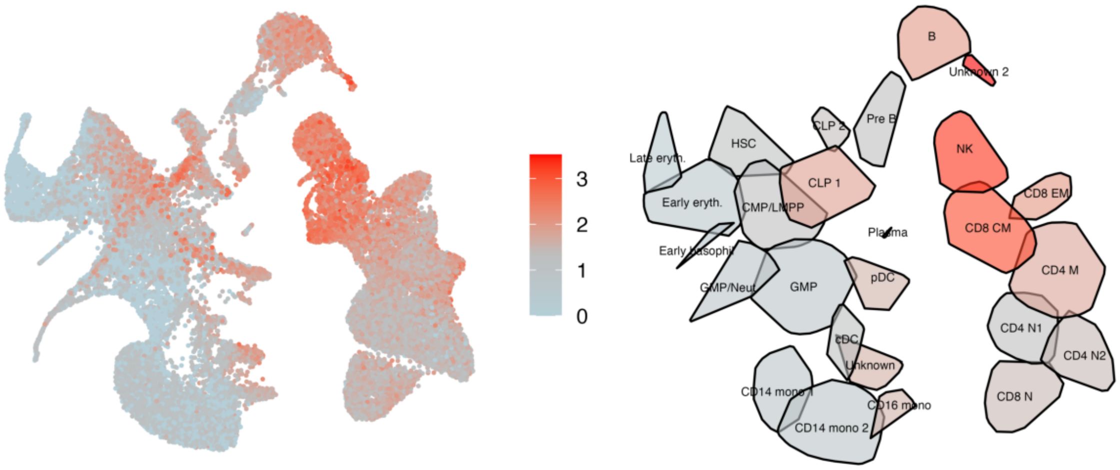

CXCR4

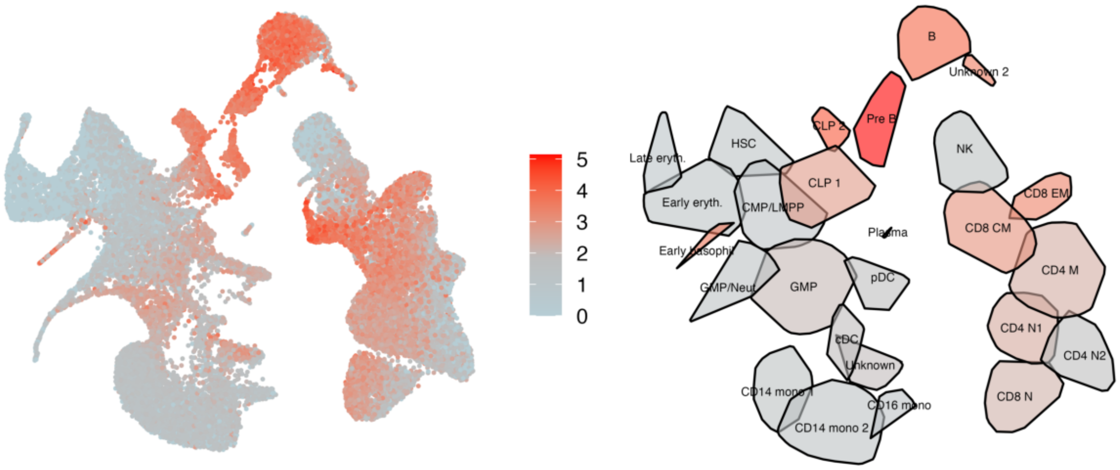

EEFSEC

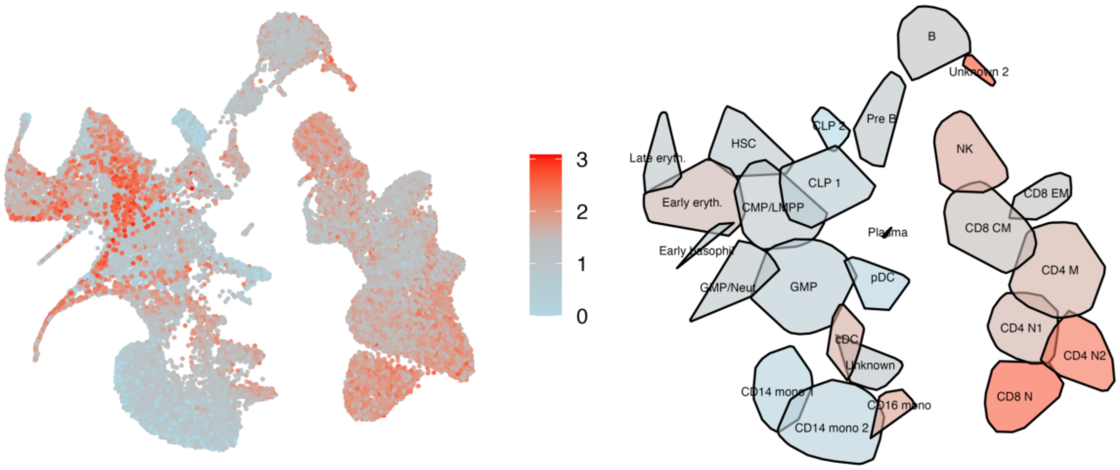

ENO1

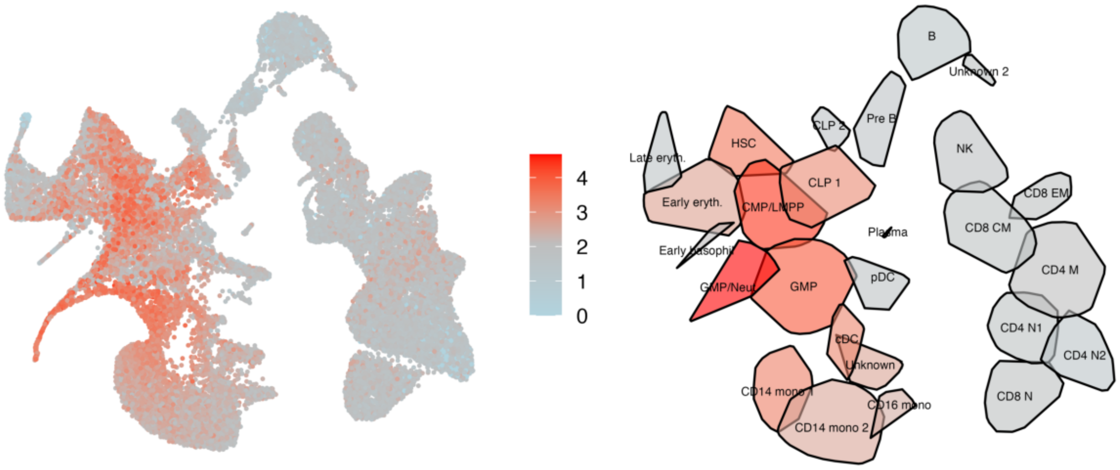

EXOC3L2

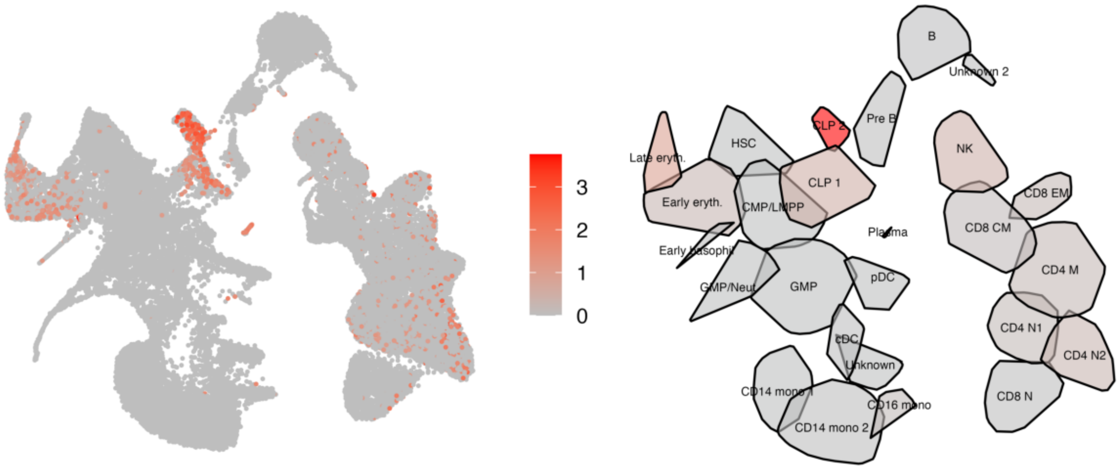

GATA2

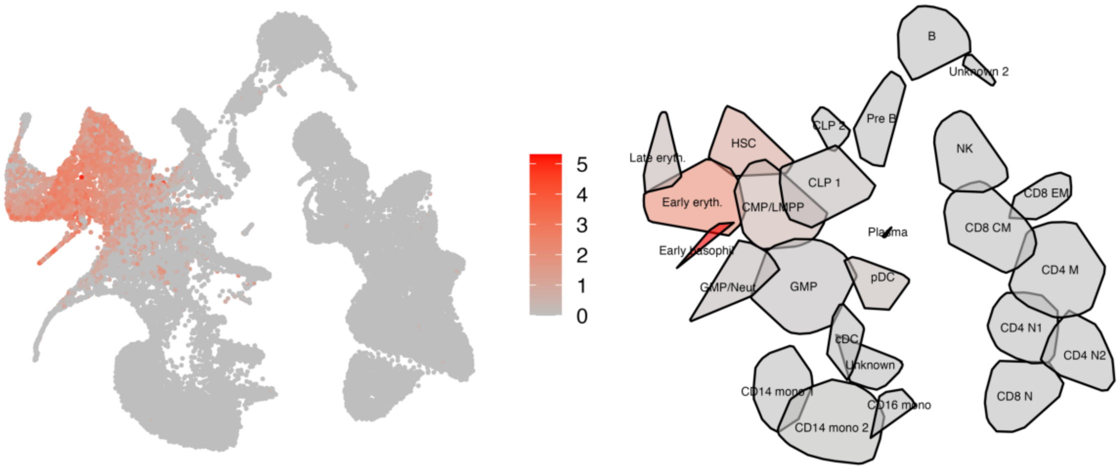

ITGA9

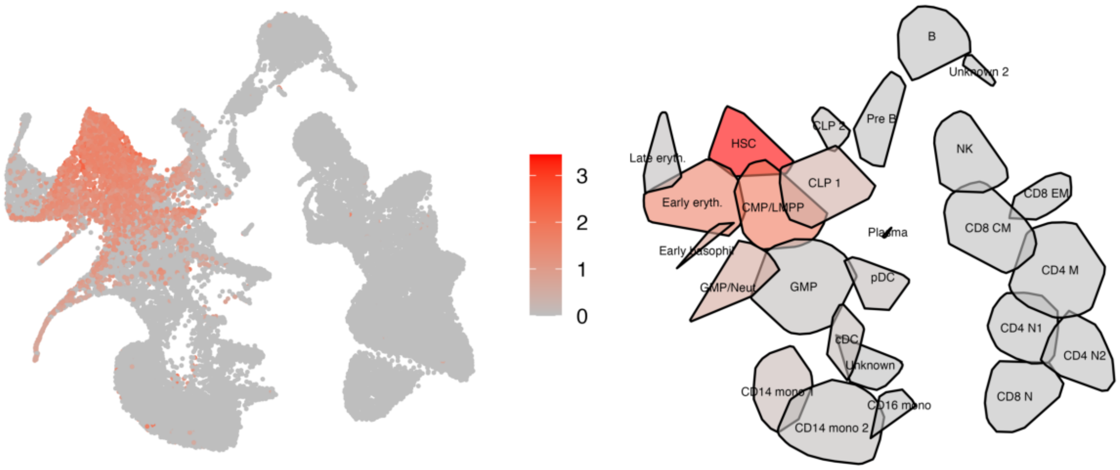

KLF2

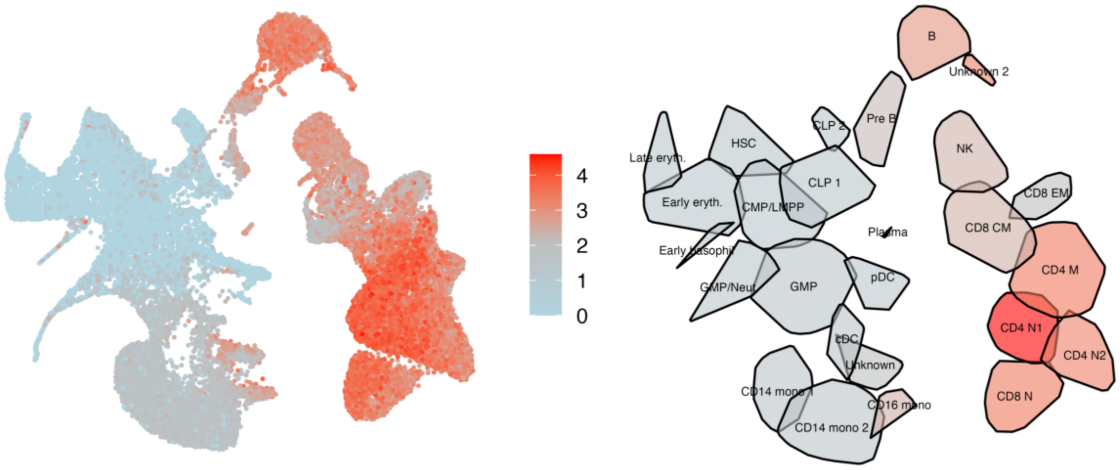

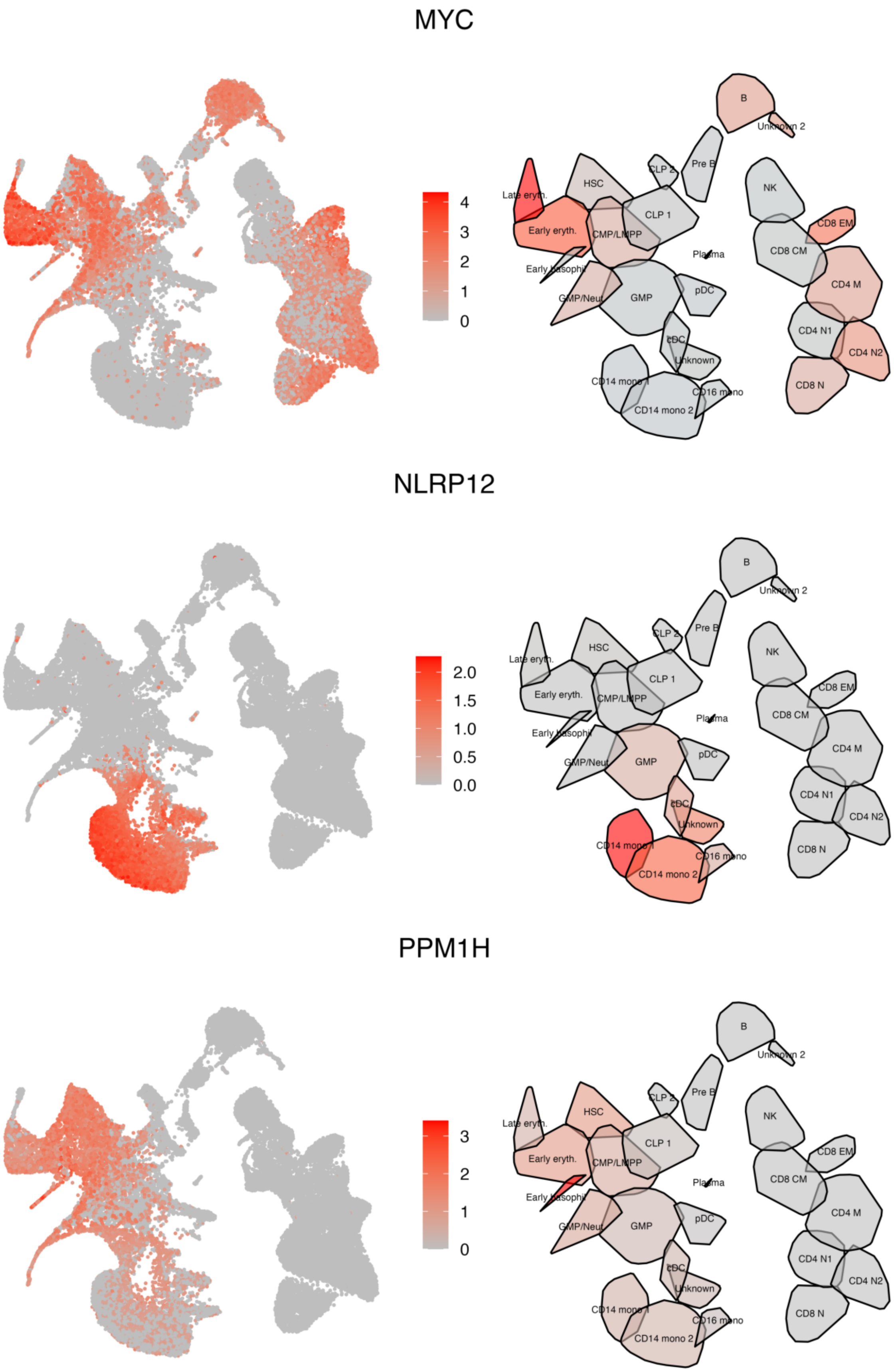
MYC
4
3
2
1
0
B
Unknown 2
Pre B
CLP 2
HSC
Late eryth.
CLP 1
NK
Early eryth.
CMP/LMPP
CD8 EM
CD8 CM
Plasma
Early basophil
GMP/Neut
GMP
pDC
CD4 M
CD4 N1
cDC
CD4 N2
Unknown
CD14 mono 1
CD16 mono
CD8 N
CD14 mono 2
NLRP12
2.0
1.5
1.0
0.5
0.0
PPM1H
3
2
1
0

PRDM16

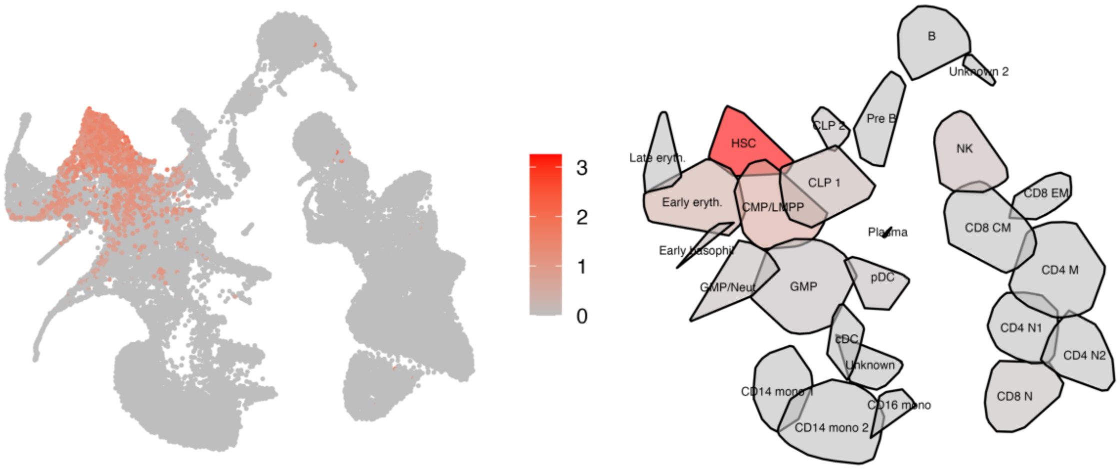

RERE

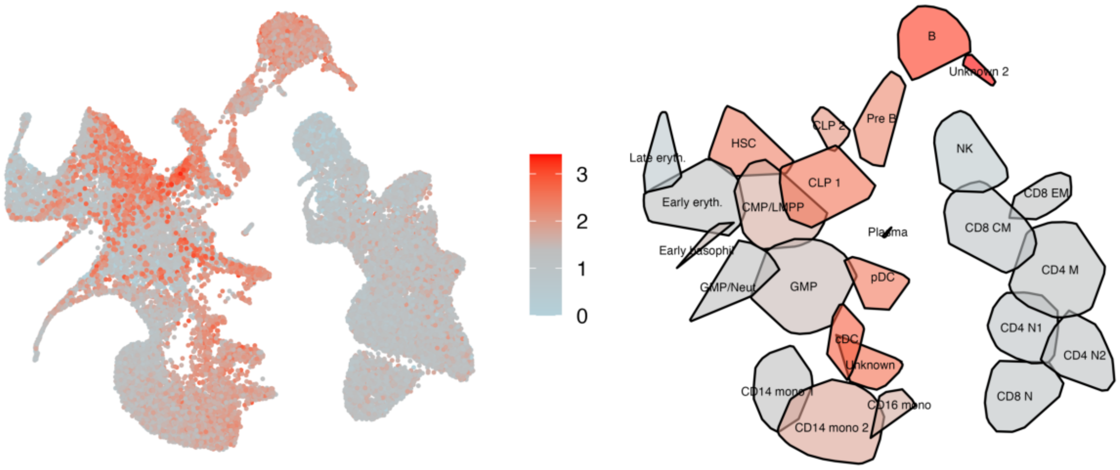

RUNX1

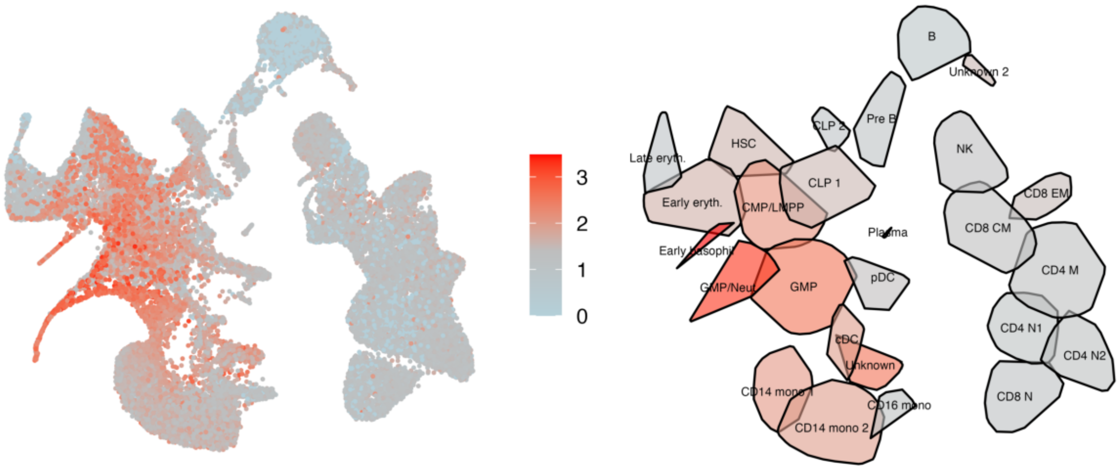

Supplementary Figure 7

Candidate gene expression in the mRNA-seq data layer from scCITE-seq analysis of 4,905 CD34$^+$ cells from adult bone marrow. Dimension-reduction using uniform manifold approximation and projection (UMAP). x- and y-axes indicate projection of cell expression patterns on the first and second UMAP components, respectively. Data are imputed expression values, shown per individual cell (left) or per cluster (right).

Cluster descriptions: **HSC-I and HSC-II:** These two HSC clusters combined constituted more than 60% of surface marker defined HSC. **HSC-III:** This HSC cluster had lowest MEP (see below) fate potential. This cluster constituted only 10-15% of antibody-derived tag (ADT)-defined HSCs. **MPP-I:** Multi-potential progenitors I. This is the most primitive MPP cluster. About 90% ADT-defined MPPs consisted of HSCI-III and MPPI-III. MPP-I had highest representation in the ADT-defined MPP population (about 20%). **MPP-II:** Multi-potential progenitors I. This cluster is biased towards MEP fate (see MEP-I). **MPP-III:** Multi-potential progenitors III. This MPP cluster is biased towards GMP (see GMP) fate. **Ly-I:** Lymphoid I. More than 60% of ADT-defined LMPP cells are present in this cluster. **Ly-II:** Lymphoid II. This cluster contains about 70% of ADT-defined CLPs. **GMP:** Granulocyte-Monocyte progenitors. This cluster contains more than 50% of ADT-defined GMP and CMP cells. **MB:** Bone marrow mast cell/basophil cluster. **DC-I:** Dendritic cell progenitors. **DC-II:** Dendritic cell progenitors further towards DC fate than DC-I. **MEP-I:** Early Megakaryote-Erythroid progenitors. This cluster contains more than 50% of ADT-defined MEPs. **MEP-II:** This cluster is composed of cells that have further lineage-committed than MEP-I. **Cyc-I and II:** These clusters are cycling cells that are not in G1 phase.

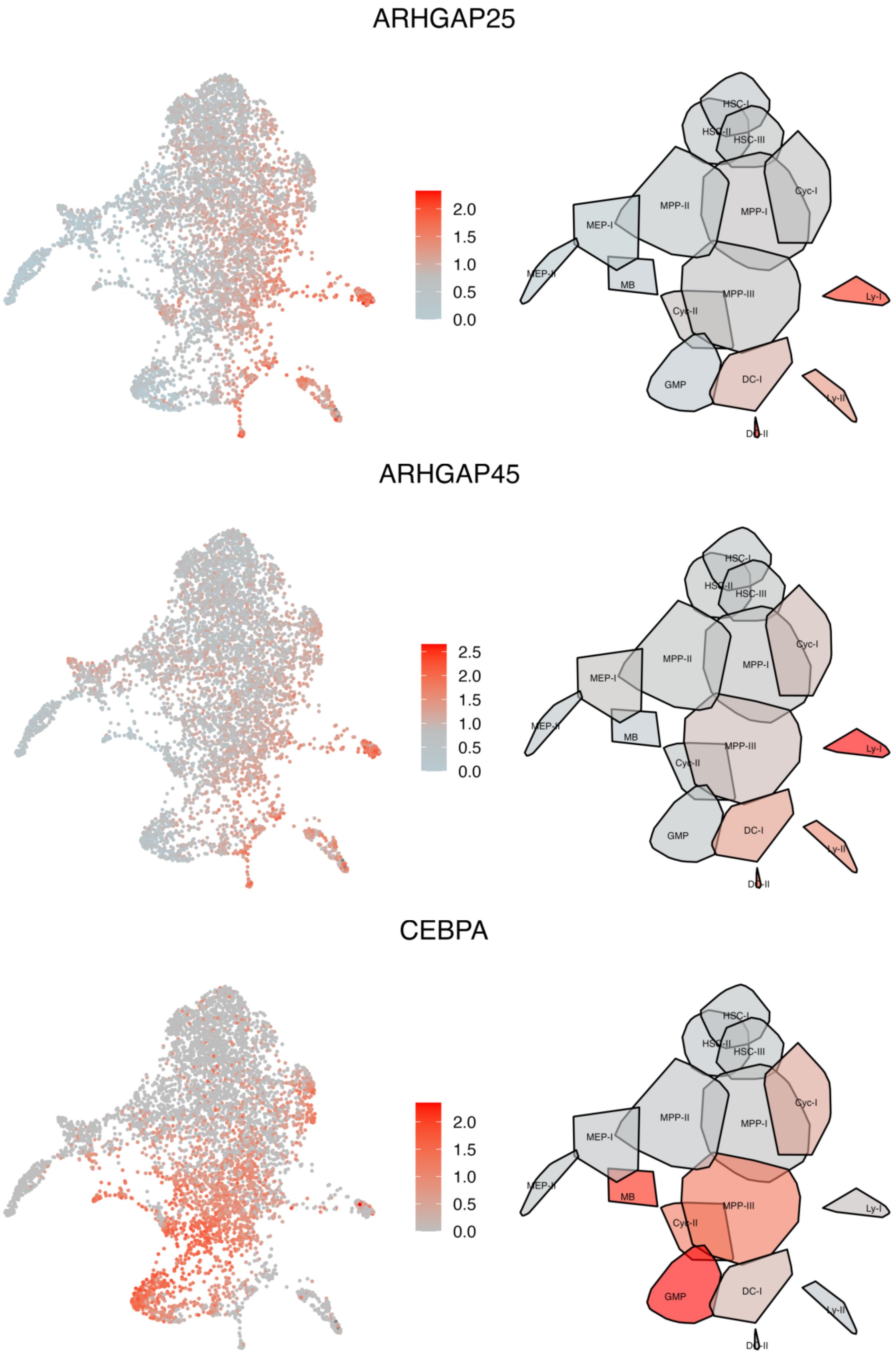

ARHGAP25
2.0
1.5
1.0
0.5
0.0
HSC-I
HSC-II
HSC-III
Cyc-I
MPP-II
MPP-I
MEP-I
MEP-II
MB
MPP-III
Ly-I
Cyc-II
GMP
DC-I
Ly-II
DC-II
ARHGAP45
2.5
2.0
1.5
1.0
0.5
0.0
CEBPA
2.0
1.5
1.0
0.5
0.0

CXCR4

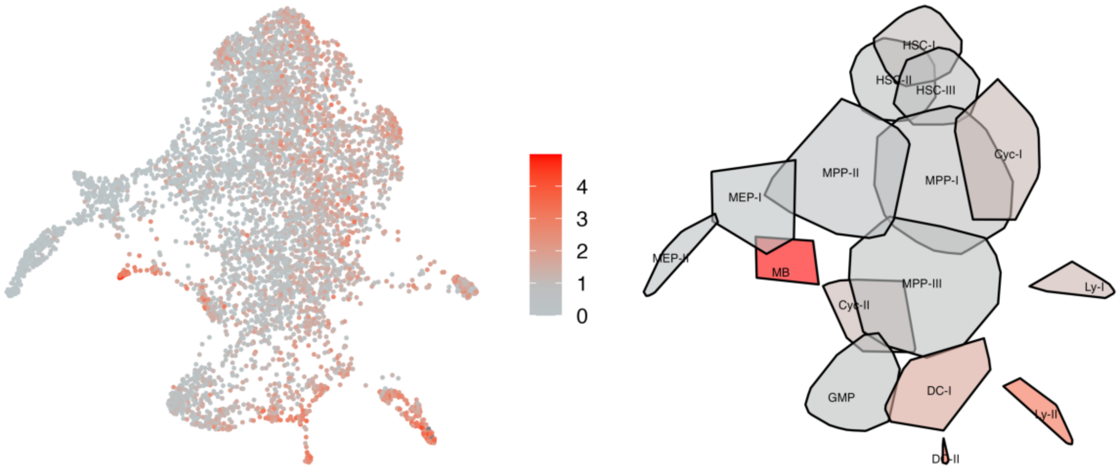

EEFSEC

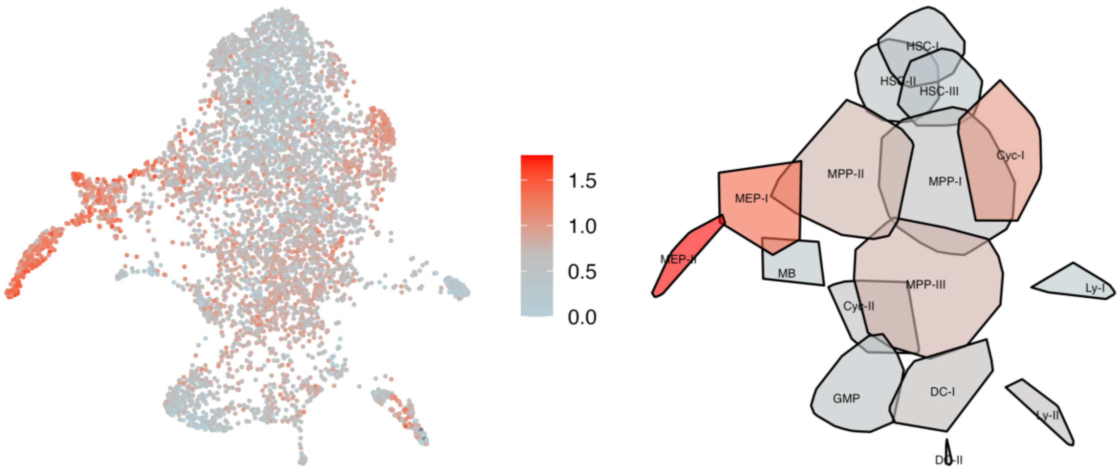

ENO1

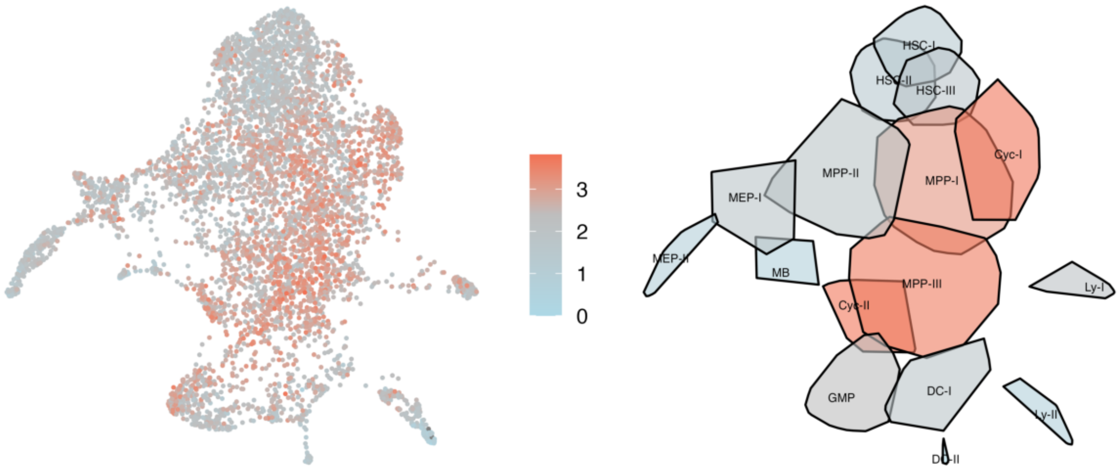

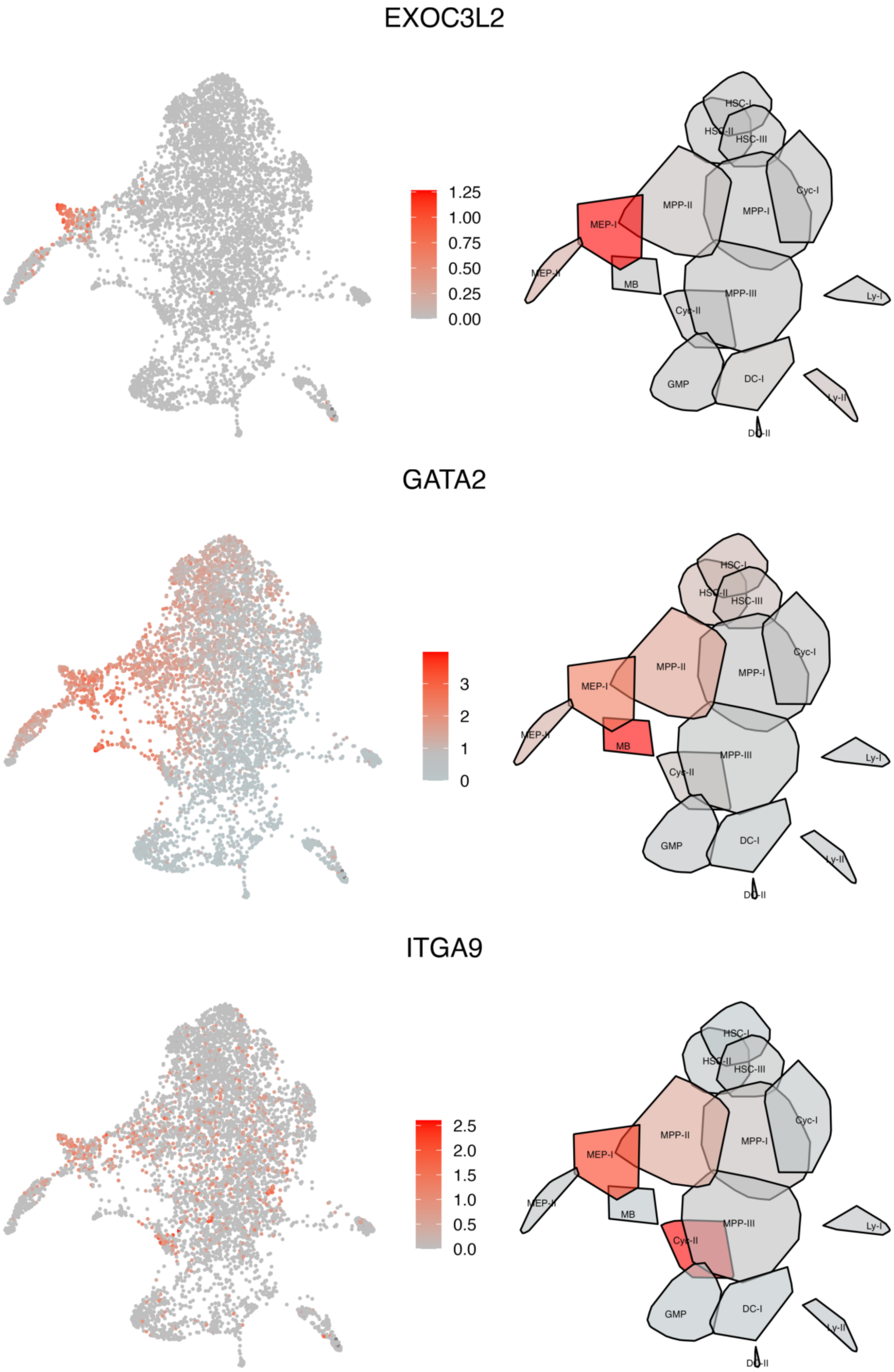
EXOC3L2
1.25
1.00
0.75
0.50
0.25
0.00
HSC-I
HSC-II
HSC-III
Cyc-I
MPP-II
MPP-I
MEP-I
MEP-II
MB
MPP-III
Ly-I
Cyc-II
GMP
DC-I
Ly-II
DC-II
GATA2
3
2
1
0
HSC-I
HSC-II
HSC-III
Cyc-I
MPP-II
MPP-I
MEP-I
MEP-II
MB
MPP-III
Ly-I
Cyc-II
GMP
DC-I
Ly-II
DC-II
ITGA9
2.5
2.0
1.5
1.0
0.5
0.0
HSC-I
HSC-II
HSC-III
Cyc-I
MPP-II
MPP-I
MEP-I
MEP-II
MB
MPP-III
Ly-I
Cyc-II
GMP
DC-I
Ly-II
DC-II

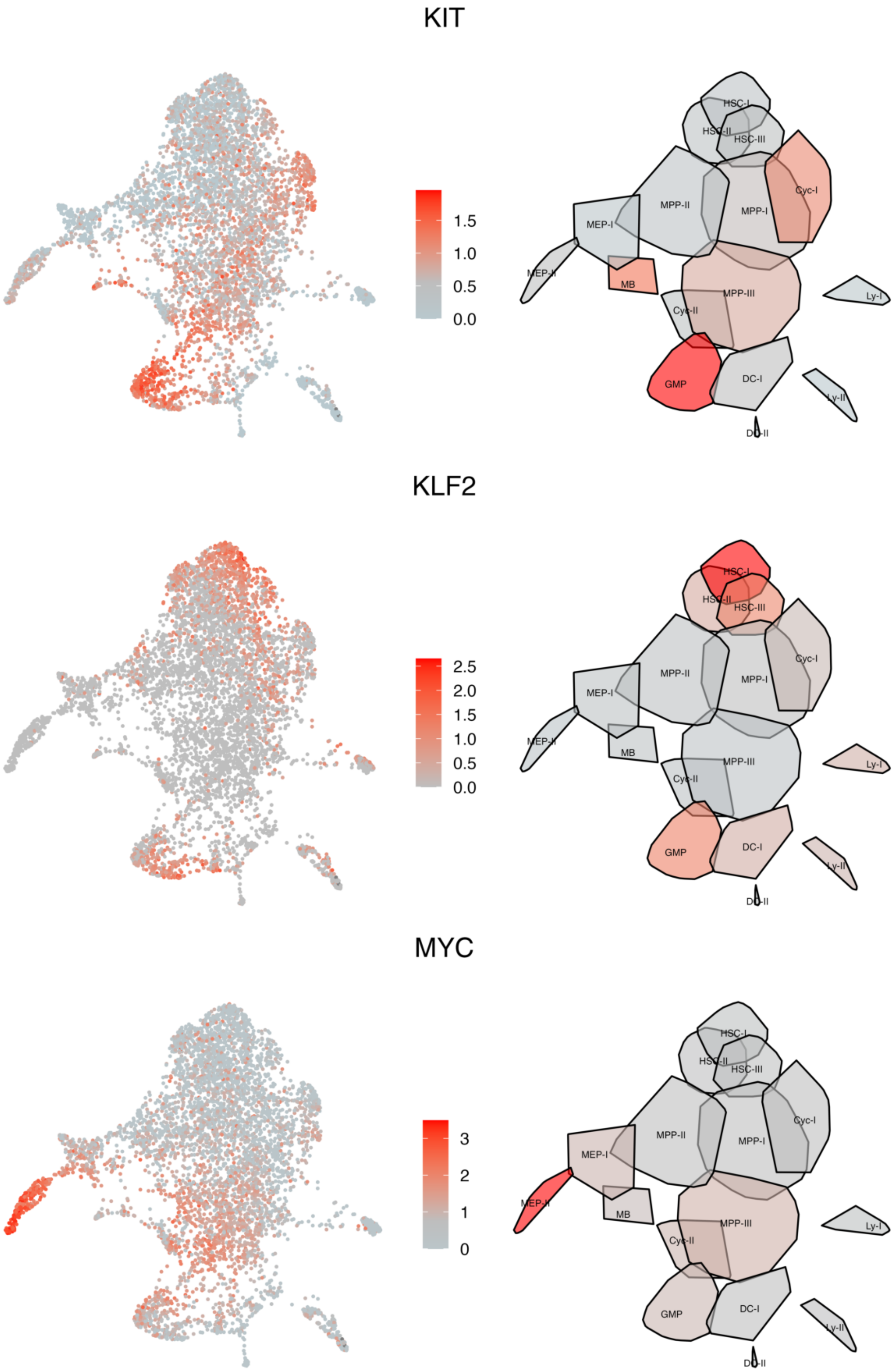
KIT
1.5
1.0
0.5
0.0
HSC-I
HSC-II
HSC-III
Cyc-I
MPP-II
MPP-I
MEP-I
MEP-II
MB
MPP-III
Ly-I
Cyc-II
GMP
DC-I
Ly-II
DC-II
KLF2
2.5
2.0
1.5
1.0
0.5
0.0
HSC-I
HSC-II
HSC-III
Cyc-I
MPP-II
MPP-I
MEP-I
MEP-II
MB
MPP-III
Ly-I
Cyc-II
GMP
DC-I
Ly-II
DC-II
MYC
3
2
1
0
HSC-I
HSC-II
HSC-III
Cyc-I
MPP-II
MPP-I
MEP-I
MEP-II
MB
MPP-III
Ly-I
Cyc-II
GMP
DC-I
Ly-II
DC-II

NLRP12

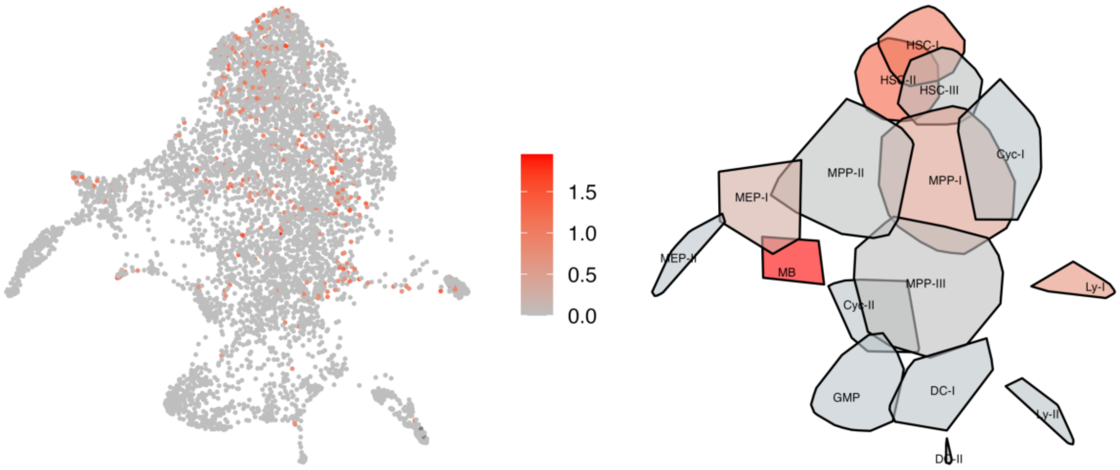

PPM1H

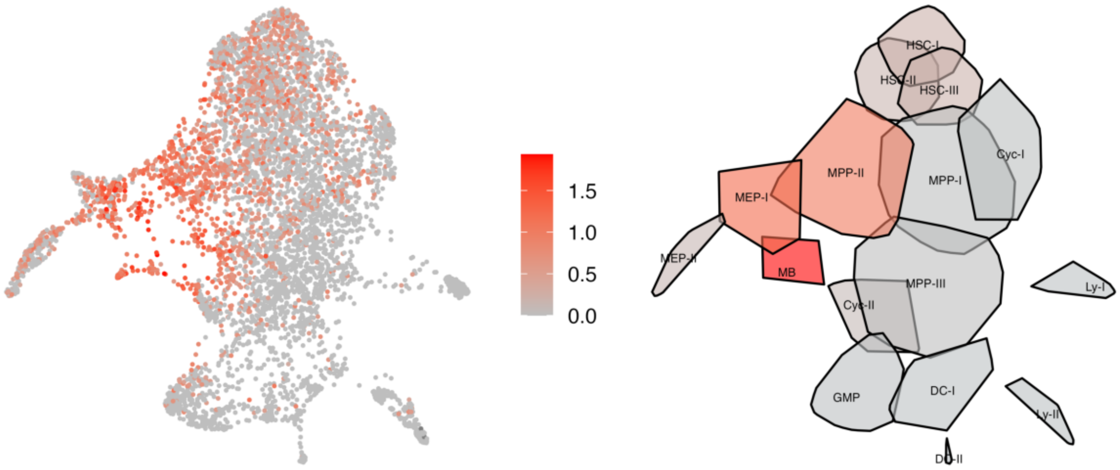

PRDM16

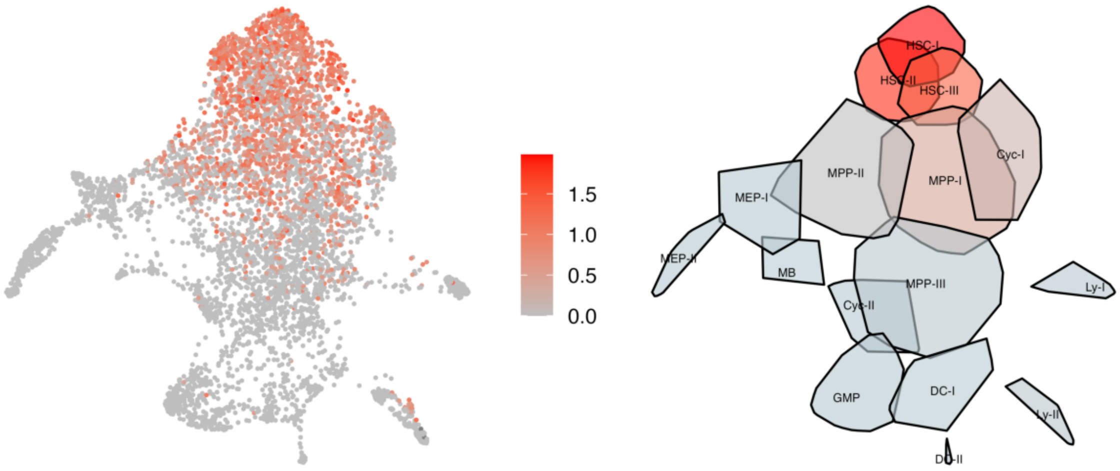

RERE

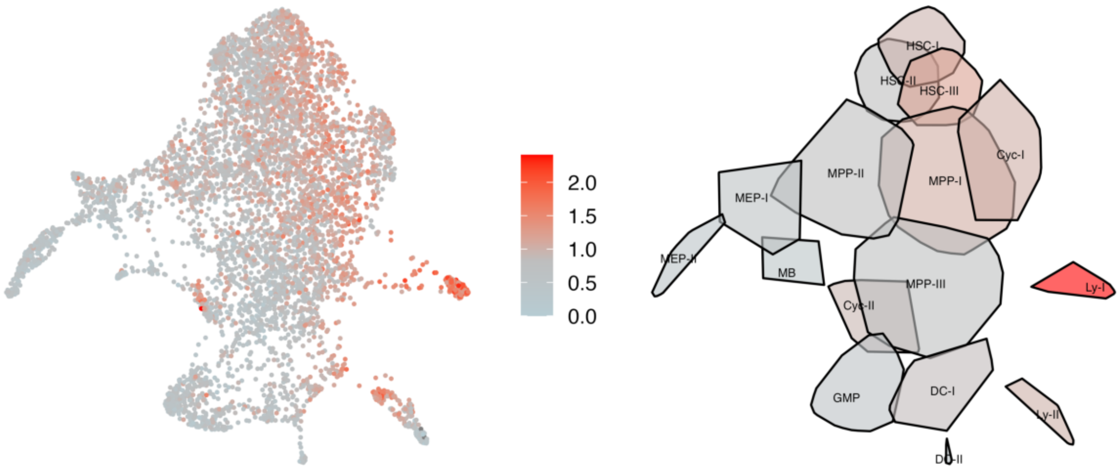
2.0
1.5
1.0
0.5
0.0
HSC-I
HSC-II
HSC-III
Cyc-I
MPP-II
MPP-I
MEP-I
MEP-II
MB
MPP-III
Ly-I
Cyc-II
GMP
DC-I
Ly-II
DC-II

RUNX1

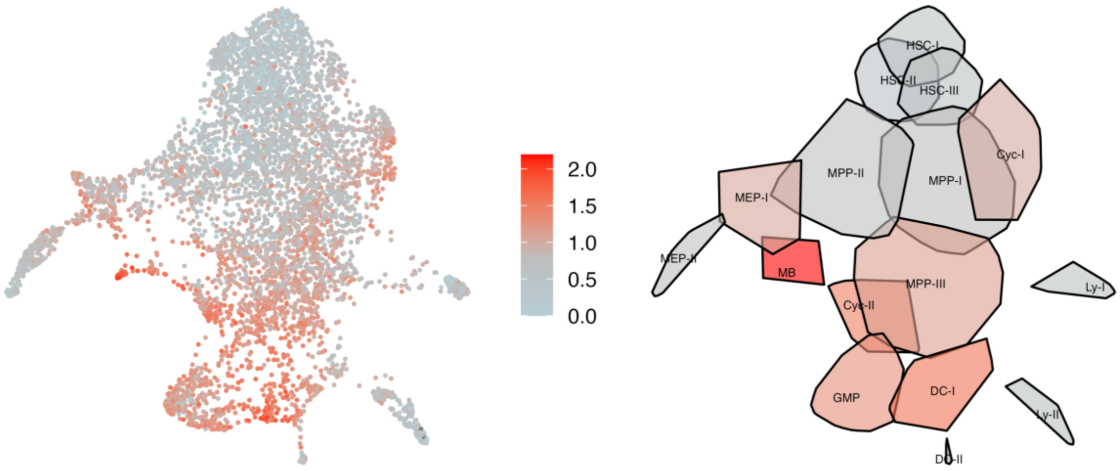
2.0
1.5
1.0
0.5
0.0
HSC-I
HSC-II
HSC-III
Cyc-I
MPP-II
MPP-I
MEP-I
MEP-II
MB
MPP-III
Ly-I
Cyc-II
GMP
DC-I
Ly-II
DC-II
