# Supplementary material for: Genome‐wide association study in 21,271 individuals identifies 9 novel loci associated with circulating CD34+ hematopoietic stem and progenitor cell levels
Source: Hemasphere. 2026 Jul 1;10(7):e70416. doi: 10.1002/hem3.70416 (PMC13321468; doi:10.1002/hem3.70416)
Supplement: Supplementary file 3 — Supplementary Information. [file HEM3-10-e70416-s003.docx]

**SUPPLEMENTARY METHODS**

**Study population**

We collected 13,470 samples from primary care patients in the Lund area of southern Sweden (“Phase IV”; **Supplementary Table 1** and **Supplementary Fig. 1**). In addition, we included blood donors and primary care patients from our previous genome-wide association study (“Phase I to III”)^1^. Blood donor samples were collected with informed consent at the Clinical Immunology and Transfusion Medicine department, Skåne University Hospital. Primary care samples consisted of surplus material from the Clinical Chemistry department at Skåne University Hospital. By programming sample processing robots at Clinical Chemistry, samples from patients aged 18 to 71 years from primary care clinics were automatically selected. Samples were annotated with age and irreversibly anonymized before being transferred to our lab. The study was approved by the Lund University Ethical Review Board (no. 2018/2) and conducted in accordance with the Declaration of Helsinki. The samples were collected as part of the BloodVariome (European Research Council project no. CoG-770992), aiming to map the genetic architecture of human blood and immune cells at high resolution.

**Flow-cytometry phenotyping**

Red blood cells were lysed with 0.84% ammonium chloride for 10 minutes, followed by centrifugation at 1200 rpm for 5 minutes. The leukocyte pellet was washed twice with 20 ml washing buffer (PBS with 4 mM EDTA). Subsequently, 80 µl of cells were transferred to a 96-well plate and centrifuged at 1200 rpm for 1 minute. Cells were stained with 30 µl of antibody cocktail (**Supplementary Table 2**), containing anti-CD45-APC-H7 (clone 2D1; BD #560178) and either anti-CD34-PerCP-Cy5.5 (clone 8G12, BD #347222; Phases I and II) or anti-CD34-PE-CF594 (clone 563, BD #562449; Phase III and IV) for 15 min at room temperature. Samples were analyzed within 36 hours of blood draw using a Bio-Rad ZE5^TM^ flow cytometer equipped with 488, 650, 405, and 561 nm lasers, as well as an autosampler. To ensure the robust quantification of rare CD34^+^ cells, we applied a high sampling depth (up to 1 million cells/sample, with a median of 562,688 in Phase IV; **Supplementary Table 3**).

**Flow-cytometry data analysis**

The gating strategy is illustrated in **Supplementary Fig. 2**. Briefly, single cells were identified using forward scatter area (FSC-A) *vs* forward scatter height (FSC-H). Peripheral blood mononuclear cells (PBMCs) were then gated based on forward scatter area *vs* side scatter area (SSC-A). CD45^+^ and CD34^+^CD45^low^ cells were subsequently gated based on their fluorescence intensity for CD34 and CD45. Blood CD34^+^ cell frequency was calculated as the number of CD34^+^ CD45^low^ cells divided by the number of CD45^+^ mononuclear cells^1^.

To facilitate the analysis of the large volumes of flow cytometry data, we used computer-assisted gating with pattern recognition algorithms implemented in AliGater^2^ (<https://github.com/LudvigEk/AliGater> and <https://github.com/LudvigEk/HSPC-regulators-in-human-blood>). Briefly, to gate singlets in FSC-A *vs* FSC-H space, we used an ellipsoid gate defined using principal component analysis (**Supplementary Fig. 2a**). To separate PBMCs and granulocytes in FSC-A *vs* SSC-A space, we used Dijkstra’s shortest-path algorithm to find the path from the left edge (minimal FSC-A) to the right edge (maximal FSC-A), yielding the lowest total event density. The PBMC gate was then refined by overlaying an ellipsoid gate defined by PCA (**Supplementary Fig. 2b**). In CD34 *vs* CD45 space, debris and CD45^+^ cells were separated by finding the CD45 intensity value corresponding to the lowest event density among CD34^-^ events, and CD34 and CD45 intensity boundaries were inferred for the CD34^+^45^low^ cluster (**Supplemental Fig. 2c**). All automated gating results were manually reviewed for additional quality control.

**Genotyping and association analysis**

Samples were genotyped using Illumina OmniExpress-24 and Global Screening single-nucleotide polymorphism (SNP) microarrays. Samples and variants with <98% yield were excluded. For samples in Phases I to III, the phasing of the chip genotypes was performed in conjunction with 91,727 samples from Sweden using Eagle2^3^. Imputation was done using a phased reference panel of whole-genome sequence data from 50,839 European-ancestry individuals, including 3,697 Swedish individuals, sequenced using HiSeqX and NovaSeq PCR-free Illumina technology to a mean depth of at least 30X^4^.

Single-nucleotide polymorphisms (SNPs) and insertions/deletions (indels) were called using Graphtyper^5^ and imputed into the phased chip genotypes. The population structure was analysed using ADMIXTURE v1.23 (<http://www.genetics.ucla.edu/software>)^6^ in supervised mode with 1000 Genomes populations CEU, CHB, and YRI as training samples^7^ and Swedish individuals as test samples. Samples with less than 0.9 CEU ancestry were excluded. To identify and exclude individuals of Finnish/Saami origin, we included individuals with less than 0.3 CHB (Asian) and less than 0.05 YRI (African) ancestry. The remaining samples were projected onto 20 principal components calculated with a European reference panel. UMAP (<https://github.com/diazale/umap_review>)^8^ was used to reduce the coordinates of test samples to two dimensions. Additional European samples not in the original reference set were also projected onto the principal components and UMAP components to identify ancestries, and samples with Swedish ancestry were identified. The inclusion of putatively Finnish/Saami individuals allowed us to confirm that we could identify a distinct UMAP cluster of individuals with the following properties: **(a)** elevated CHB (Asian) ancestry according to ADMIXTURE; **(b)** enriched for individuals who we knew to have been born in Finland; **(c)** on principal component 1 and 2, were positioned in the region occupied by Finnish individuals. Those individuals were excluded from the analysis. After exclusion, Phases I-III included 11,644 samples classified as Swedish.

In Phase IV, chip genotypes were phased and imputed using SHAPEIT v2.790^9^ and IMPUTE2 v2.3.2^10^ using 1000 Genomes Phase 3 (October 2014), which includes phased genotypes for ~80 million variants for 2,504 individuals^7^. The population structure of Phase IV samples was analyzed using ADMIXTURE v1.23^6^ in supervised mode using the 1000 Genomes populations of European, African, and East Asian ancestry for training. Samples assigned > 0.06 African or East Asian ancestry were excluded. Principal components were calculated for 9,627 samples identified as Swedish using EIGENSOFT v 6.0.1^11^ and used to adjust for the remaining population structure.

Association testing was performed separately in Phases I-III and Phase IV. Prior to association testing, the blood CD34^+^ cell levels were adjusted for sex, age, twelve principal components, and phase, and subsequently standardized using an inverse normal transform. For Phases I-III, the association test was performed using a generalized form of linear regression, which estimated the effects of genotypes on adjusted and standardized blood CD34^+^ cell levels using an additive model^12^. For Phase IV, we used linear regression in SNPTEST v.2.5^13^ using an additive model and a likelihood score test. Linkage disequilibrium (LD) score regression was used to account for distribution inflation in the association test statistics due to cryptic relatedness and population stratification^14^. The results for 32 million variants from the two sets were combined using a fixed-effects inverse-variance meta-analysis^15^. To account for multiple testing, we applied a class-based Bonferroni correction, grouping variants by genomic annotation and adjusting the significance threshold for each category based on the number of variants (**Supplementary Table 4**)^16^. A standard whole-genome Bonferroni correction assumes equal prior probability of causality across all variants. However, certain classes of variants (e.g., loss-of-function or missense variants) are more likely to alter gene function than others (*e.g*., synonymous or intergenic variants). The weighted Bonferroni framework accounts for this by assigning different weights based on functional annotation, thereby maintaining strict control of the family-wise error rate while increasing power to detect associations in biologically relevant regions. Stepwise conditional analysis was used to identify independent signals in each associated region. For each signal, the 99% credible set of likely causal variants was defined using Bayesian refinement^17^.

**Heritability estimation**

To calculate the total variance explained (*h*^2^) by the identified variants, we used the formula *h*^2^​ = ∑ 2 × β_i_^2^ × MAF_i_​ × (1 − MAF_i​_), where MAF_i_ and β_i_ denote the minor allele frequency and effect size for the *i*:th lead variant. Total SNP heritability was estimated using linkage disequilibrium score regression ^14,18^. To assess cell type-specific partitioned heritability based on chromatin accessibility, we used LD-scores derived from ATAC-seq data for sorted blood cell types available for LDSC^14^, supplemented with LD-scores for myeloid and plasmacytoid dendritic cells computed from published ATAC-seq data (NCBI Gene Expression Omnibus accession no. GSE119453)^19^**.**

**Expression quantitative trait locus (eQTL) analysis in CD34^+^ cells**

To assess the effects of the identified variants on gene expression in CD34^+^ cells, we analyzed our previously generated mRNA-sequencing data for CD34^+^ cells purified from the peripheral blood of routine blood donors (European Genome Archive repository (accession no. EGAS00001005655 and EGAD00001008194)^1^. Briefly, we purified CD34^+^ cells from leukocyte filters (Reveos^TM^) from 155 routine blood donors. We purified CD34^+^ cells (average 122,000 per sample) using immunomagnetic beads (MACSprep CD34^+^ MicroBead kit; Miltenyi Biotech #130-120-673) followed by fluorescence-activated cell sorting with anti-human CD34 (clone 563; BD #562449) and anti-CD4 (clone 2D1; BD #560178). mRNA from purified cells was sequenced on an Illumina NovaSeq 6000 sequencer with a read length of 100 bases in paired-end mode. Raw gene counts were transformed to FPKM values. To test associations between variants and gene expression, we used linear modeling with the variant genotype as the independent variable and ten principal component covariates, calculated using genes with an average FPKM value greater than 1.0 in our dataset.

**Chromatin accessibility analysis**

To identify putative causal gene-regulatory variants within credible sets, we used published ATAC-seq data for sorted blood cell types (UCSC Genome Browser hub <https://atac-blood-hg38.s3.amazonaws.com/hub.txt>), including CD34^+^ subpopulations^19^. To test for enrichment of association signal within open chromatin of specific blood cell types, we used the linkage disequilibrium score regression (LDSC)^14^ and g-chromVAR^19^.

**Chromatin looping analysis**

To identify candidate causal variants that interact with gene promoters through chromatin looping interactions, we used PCHi-C (promoter capture Hi-C) data for peripheral blood CD34^+^ cells (ArrayExpress accession number E-MTAB-2323)^20^. These data had been previously processed using the GOTHiC Bioconductor package^21^ and filtered to interactions with a false discovery rate < 0.05 in two biological replicates. Data were lifted over to hg38 with CrossMap^22^. The log of the observed/expected ratio shown in locus plots is an estimate of effect size or interaction frequency. Genes whose promoters were linked to restriction fragments harboring credible set variants were assigned to those credible sets.

**Analysis of candidate gene expression in hematopoietic cell types**

To map the expression patterns of candidate genes in hematopoietic cell types, we analyzed single-cell mRNA sequencing data (scRNA-seq) from 35,882 mononuclear cells derived from blood and bone marrow of four healthy donors (Gene Expression Omnibus accession GSE139369)^23^, alongside bulk mRNA sequencing data from sorted blood cells (GSE96772 and GSE74912)^24,25^ . To test for enrichment of expression in HSPCs, we compared the median-centered log_2_ ratios in CD34^+^ vs other cell types using the Wilcoxon ranksum test.

To examine gene expression within the CD34^+^ cell compartment, we utilized single-cell CITE-seq (Cellular Indexing of Transcriptomes and Epitopes by sequencing) data from 4,905 CD34^+^ cells isolated from bone marrow (GSE173076)^26^. The definitions of the cell populations in these data sets are described in the original publications. For dimension reduction of single-cell data, we used uniform manifold approximation and projection (UMAP)^27^, and imputed gene expression using ALRA^28,29^ .Plots were generated using Seurat^30^. Bulk mRNA-sequencing data and bulked single-cell data were visualized as log_2_-transformed expression values, median-centered across cell types within each data set.

**Genetic overlap with other traits and diseases**

To assess genetic overlap between the identified variants and previously reported variants associated with diseases and other quantitative traits, we queried the GWAS catalog using the LDTrait webtool (<https://ldlink.nci.nih.gov/?tab=ldtrait>), with default settings and a linkage disequilibrium threshold of *r*^2^ > 0.8 in Europeans^31,32^. Additionally, we queried release 12 of the FinnGen database (<https://r12.finngen.fi>). Genetic correlations were calculated using LDSC using summary statistics from the UK Biobank.

To investigate the overlap with recurrently somatically mutated genes in different tumor types, we considered genes designated as driver genes in the IntOGen database (https://www.intogen.org)^33^ as well as fusion gene partners recurrently reported (five times or more) in the Mitelman database ([https://mitelmandatabase.isb-cgc.org](https://mitelmandatabase.isb-cgc.org/))^34^. To test for enrichment, we used binomial test as an approximation to a hypergeometric test because the background population is large compared to the sample, the tested set is a small set of the background population, and the expected probability is fixed. To assess the essentiality of candidate genes to the growth of cancer cell lines derived from different types of malignancies, we used DepMap version 24Q4 (<https://depmap.org>)^35^.

**REFERENCES**

1 Lopez de Lapuente Portilla A, Ekdahl L, Cafaro C, Ali Z, Miharada N, Thorleifsson G *et al.* Genome-wide association study on 13 167 individuals identifies regulators of blood CD34+cell levels. *Blood* 2022; **139**. doi:10.1182/blood.2021013220.

2 Ekdahl L, Arrizabalaga AL, Ali Z, Cafaro C, Lopez de Lapuente Portilla A, Nilsson B. AliGater: a framework for the development of bioinformatic pipelines for large-scale, high-dimensional cytometry data. *Bioinformatics Advances* 2023; **3**. doi:10.1093/bioadv/vbad103.

3 Loh PR, Palamara PF, Price AL. Fast and accurate long-range phasing in a UK Biobank cohort. *Nat Genet* 2016; **48**. doi:10.1038/ng.3571.

4 Gudbjartsson DF, Helgason H, Gudjonsson SA, Zink F, Oddson A, Gylfason A *et al.* Large-scale whole-genome sequencing of the Icelandic population. *Nat Genet* 2015; **47**. doi:10.1038/ng.3247.

5 Eggertsson HP, Jonsson H, Kristmundsdottir S, Hjartarson E, Kehr B, Masson G *et al.* Graphtyper enables population-scale genotyping using pangenome graphs. *Nat Genet* 2017; **49**. doi:10.1038/ng.3964.

6 Alexander DH, Novembre J, Lange K. Fast model-based estimation of ancestry in unrelated individuals. *Genome Res* 2009; **19**. doi:10.1101/gr.094052.109.

7 The 1000 Genomes Project Consortium. A global reference for human genetic variation The 1000 Genomes Project Consortium*. *Nature* 2015; **526**.

8 Diaz-Papkovich A, Anderson-Trocmé L, Gravel S. A review of UMAP in population genetics. J. Hum. Genet. 2021; **66**. doi:10.1038/s10038-020-00851-4.

9 Delaneau O, Howie B, Cox AJ, Zagury JF, Marchini J. Haplotype estimation using sequencing reads. *Am J Hum Genet* 2013; **93**. doi:10.1016/j.ajhg.2013.09.002.

10 Howie BN, Donnelly P, Marchini J. A flexible and accurate genotype imputation method for the next generation of genome-wide association studies. *PLoS Genet* 2009; **5**. doi:10.1371/journal.pgen.1000529.

11 Price AL, Patterson NJ, Plenge RM, Weinblatt ME, Shadick NA, Reich D. Principal components analysis corrects for stratification in genome-wide association studies. *Nat Genet* 2006; **38**. doi:10.1038/ng1847.

12 Benonisdottir S, Oddsson A, Helgason A, Kristjansson RP, Sveinbjornsson G, Oskarsdottir A *et al.* Epigenetic and genetic components of height regulation. *Nat Commun* 2016; **7**. doi:10.1038/ncomms13490.

13 Marchini J, Howie B, Myers S, McVean G, Donnelly P. A new multipoint method for genome-wide association studies by imputation of genotypes. *Nat Genet* 2007; **39**. doi:10.1038/ng2088.

14 Bulik-Sullivan B, Loh PR, Finucane HK, Ripke S, Yang J, Patterson N *et al.* LD score regression distinguishes confounding from polygenicity in genome-wide association studies. *Nat Genet* 2015; **47**. doi:10.1038/ng.3211.

15 Mantel N, Haenszel W. Statistical Aspects of the Analysis of Data From Retrospective Studies of Disease 1. https://academic.oup.com/jnci/article/22/4/719/900746.

16 Sveinbjornsson G, Albrechtsen A, Zink F, Gudjonsson SA, Oddson A, Másson G *et al.* Weighting sequence variants based on their annotation increases power of whole-genome association studies. *Nat Genet* 2016; **48**. doi:10.1038/ng.3507.

17 Maller JB, McVean G, Byrnes J, Vukcevic D, Palin K, Su Z *et al.* Bayesian refinement of association signals for 14 loci in 3 common diseases. *Nat Genet* 2012; **44**. doi:10.1038/ng.2435.

18 Finucane HK, Bulik-Sullivan B, Gusev A, Trynka G, Reshef Y, Loh PR *et al.* Partitioning heritability by functional annotation using genome-wide association summary statistics. *Nat Genet* 2015; **47**. doi:10.1038/ng.3404.

19 Ulirsch JC, Lareau CA, Bao EL, Ludwig LS, Guo MH, Benner C *et al.* Interrogation of human hematopoiesis at single-cell and single-variant resolution. *Nat Genet* 2019; **51**. doi:10.1038/s41588-019-0362-6.

20 Mifsud B, Tavares-Cadete F, Young AN, Sugar R, Schoenfelder S, Ferreira L *et al.* Mapping long-range promoter contacts in human cells with high-resolution capture Hi-C. *Nat Genet* 2015; **47**. doi:10.1038/ng.3286.

21 Mifsud B, Martincorena I, Darbo E, Sugar R, Schoenfelder S, Fraser P *et al.* GOTHiC, a probabilistic model to resolve complex biases and to identify real interactions in Hi-C data. *PLoS One* 2017; **12**. doi:10.1371/journal.pone.0174744.

22 Zhao H, Sun Z, Wang J, Huang H, Kocher JP, Wang L. CrossMap: A versatile tool for coordinate conversion between genome assemblies. *Bioinformatics* 2014; **30**. doi:10.1093/bioinformatics/btt730.

23 Granja JM, Klemm S, McGinnis LM, Kathiria AS, Mezger A, Corces MR *et al.* Single-cell multiomic analysis identifies regulatory programs in mixed-phenotype acute leukemia. *Nat Biotechnol* 2019; **37**. doi:10.1038/s41587-019-0332-7.

24 Buenrostro JD, Corces MR, Lareau CA, Wu B, Schep AN, Aryee MJ *et al.* Integrated Single-Cell Analysis Maps the Continuous Regulatory Landscape of Human Hematopoietic Differentiation. *Cell* 2018; **173**. doi:10.1016/j.cell.2018.03.074.

25 Corces MR, Buenrostro JD, Wu B, Greenside PG, Chan SM, Koenig JL *et al.* Lineage-specific and single-cell chromatin accessibility charts human hematopoiesis and leukemia evolution. *Nat Genet* 2016; **48**. doi:10.1038/ng.3646.

26 Sommarin MNE, Dhapola P, Safi F, Warfvinge R, Ulfsson LG, Erlandsson E *et al.* Single-Cell Multiomics Reveals Distinct Cell States at the Top of the Human Hematopoietic Hierarchy. *bioRxiv* 2021.

27 Becht E, McInnes L, Healy J, Dutertre CA, Kwok IWH, Ng LG *et al.* Dimensionality reduction for visualizing single-cell data using UMAP. *Nat Biotechnol* 2019; **37**. doi:10.1038/nbt.4314.

28 Linderman GC, Zhao J, Roulis M, Bielecki P, Flavell RA, Nadler B *et al.* Zero-preserving imputation of single-cell RNA-seq data. *Nat Commun* 2022; **13**. doi:10.1038/s41467-021-27729-z.

29 Butler A, Hoffman P, Smibert P, Papalexi E, Satija R. Integrating single-cell transcriptomic data across different conditions, technologies, and species. *Nat Biotechnol* 2018; **36**. doi:10.1038/nbt.4096.

30 Wolf FA, Angerer P, Theis FJ. SCANPY: Large-scale single-cell gene expression data analysis. *Genome Biol* 2018; **19**. doi:10.1186/s13059-017-1382-0.

31 Lin SH, Brown DW, Machiela MJ. LDtrait: An online tool for identifying published phenotype associations in linkage disequilibrium. *Cancer Res* 2020; **80**. doi:10.1158/0008-5472.CAN-20-0985.

32 Buniello A, Macarthur JAL, Cerezo M, Harris LW, Hayhurst J, Malangone C *et al.* The NHGRI-EBI GWAS Catalog of published genome-wide association studies, targeted arrays and summary statistics 2019. *Nucleic Acids Res* 2019; **47**. doi:10.1093/nar/gky1120.

33 Gonzalez-Perez A, Perez-Llamas C, Deu-Pons J, Tamborero D, Schroeder MP, Jene-Sanz A *et al.* IntOGen-mutations identifies cancer drivers across tumor types. *Nat Methods* 2013; **10**. doi:10.1038/nmeth.2642.

34 Mitelman F JB and MF (Eds. ). Mitelman Database of Chromosome Aberrations and Gene Fusions in Cancer. 2025.

35 Arafeh R, Shibue T, Dempster JM, Hahn WC, Vazquez F. The present and future of the Cancer Dependency Map. *Nat Rev Cancer* 2025; **25**: 59–73.
